# Craspase Orthologs Cleave a Nonconserved Site in Target Protein Csx30

**DOI:** 10.1021/acschembio.3c00788

**Published:** 2024-04-11

**Authors:** Sam P. B. van Beljouw, Anna C. Haagsma, Konstantinos Kalogeropoulos, Martin Pabst, Stan J. J. Brouns

**Affiliations:** † Department of Bionanoscience, Delft University of Technology, 2629 HZ Delft, Netherlands; ‡ Kavli Institute of Nanoscience, 2629 HZ Delft, Netherlands; § Department of Biotechnology and Biomedicine, 2800 Kgs. Lyngby, Denmark; ∥ Department of Biotechnology, Delft University of Technology, 2629 HZ Delft, Netherlands

## Abstract

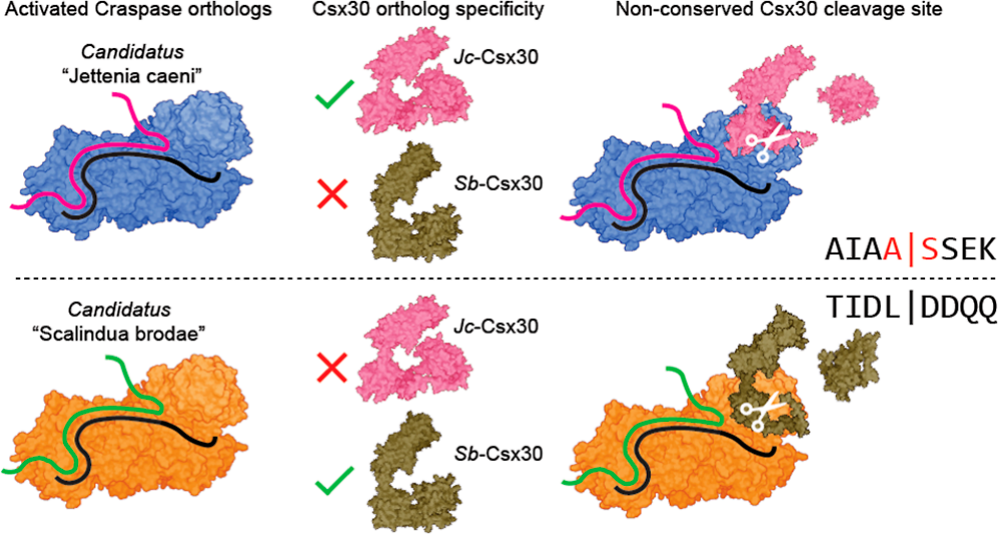

The Craspase CRISPR-Cas effector consists of the RNA-guided ribonuclease gRAMP and the protease TPR-CHAT, coupling target RNA recognition to protease activation. The natural substrate of Craspase is Csx30, a protein cleaved in two fragments that subsequently activates downstream antiviral pathways. Here, we determined the protease substrate specificity of Craspase from *Candidatus* “Jettenia caeni” (*Jc*-Craspase). We find that *Jc*-Craspase cleaves *Jc*-Csx30 in a target RNA-dependent fashion in A|S, which is different from the sites found in two other studied Craspases (L|D and M|K for *Candidatus* “Scalindua brodae” and *Desulfonema ishimotonii*, respectively). The fact that Craspase cleaves a nonconserved site across orthologs indicates the evolution of specific protein interactions between Craspase and its respective Csx30 target protein. The Craspase family thus represents a panel of proteases with different substrate specificities, which we exploited for the development of a readout for multiplexed RNA detection.

The discovery of Craspase revealed a novel feature in the CRISPR-Cas family: the coupling of sequence-specific nucleic acid detection to protease activity, all within the same effector complex. Binding of target RNA to Craspase relays a conformational change to activate the protease, which in turn cleaves CRISPR-associated protein Csx30 in a specific position.^1−4^ Csx30 is part of a complex together with Csx31 and RpoE,^3^ two proteins that are often encoded in type III-E CRISPR-Cas loci.^5,6^ In transplanted *Escherichia coli* cells, cleavage of Csx30 in the Cx30-Csx31-RpoE complex instigates transcriptional pathways via the action of liberated RpoE^4^ and induces cellular suicide via an unknown process to protect against phage infection.^3^ Despite the bioinformatic prediction of at least ten Craspase orthologs,^6^ only two—the Craspases from *Candidatus* “Scalindua brodae” (*Sb*-Craspase)^1^ and *Desulfonema ishimotonii* (*Di*-Craspase)^2^—have been experimentally described at the protease level.

Here, we set out to characterize Craspase from *Candidatus* “Jettenia caeni” (*Jc*-Craspase), an anaerobic ammonium-oxidizing bacterium that plays important roles in the global cycling of nitrogen in marine environments.^7^*Jc*-Craspase cleaves *Jc*-Csx30 only in the presence of a target RNA (Figure 1A), in line with observations from the studied Craspase orthologs.^1,2^ Mass spectrometry (MS) on the *Jc*-Csx30 protein fragments revealed the processing site between alanine (A434) and serine (S435) (Figure 1B,C), which is completely different from the other known Craspase cleavage sites (L|D for *Sb*-Craspase^1,3^ and M|K for *Di*-Craspase^2,4^). This is remarkable given the close phylogenetic relatedness of the gRAMP orthologs, especially those from *Candidatus* “S. brodae” and *Candidatus* “J. caeni”.^6^ So contrary to other proteases, which almost always display strong conservation in recognition sites across species,^8,9^ Craspase orthologs appear to cleave at a nonconserved site near the C-terminal of target protein Csx30 (Figure 1D). Individual amino acid substitutions of the eight residues surrounding the cleavage site of *Jc*-Csx30 (P4–P4’: AIAA|SSEK) were cleaved with a similar efficiency by *Jc*-Craspase compared to wild-type *Jc*-Csx30 (Figure 1E). These findings strengthen the hypothesis that structural positioning, rather than the identity of the amino acids surrounding the cleavage site, is important for Craspase protein cleavage. While mutational analysis of *Sb*-Csx30 and *Di*-Csx30 similarly did not reveal Csx30 residues that are absolutely essential for cleavage,^1,4^ some substitutions (especially in P1) were found to mildly^1,4^ or severely^2,3,10^ affect the protease activity in the studied timeframes. This implies that the amino acids constituting the Csx30 cleavage site may vary in importance among different orthologs.

**Figure 1:**
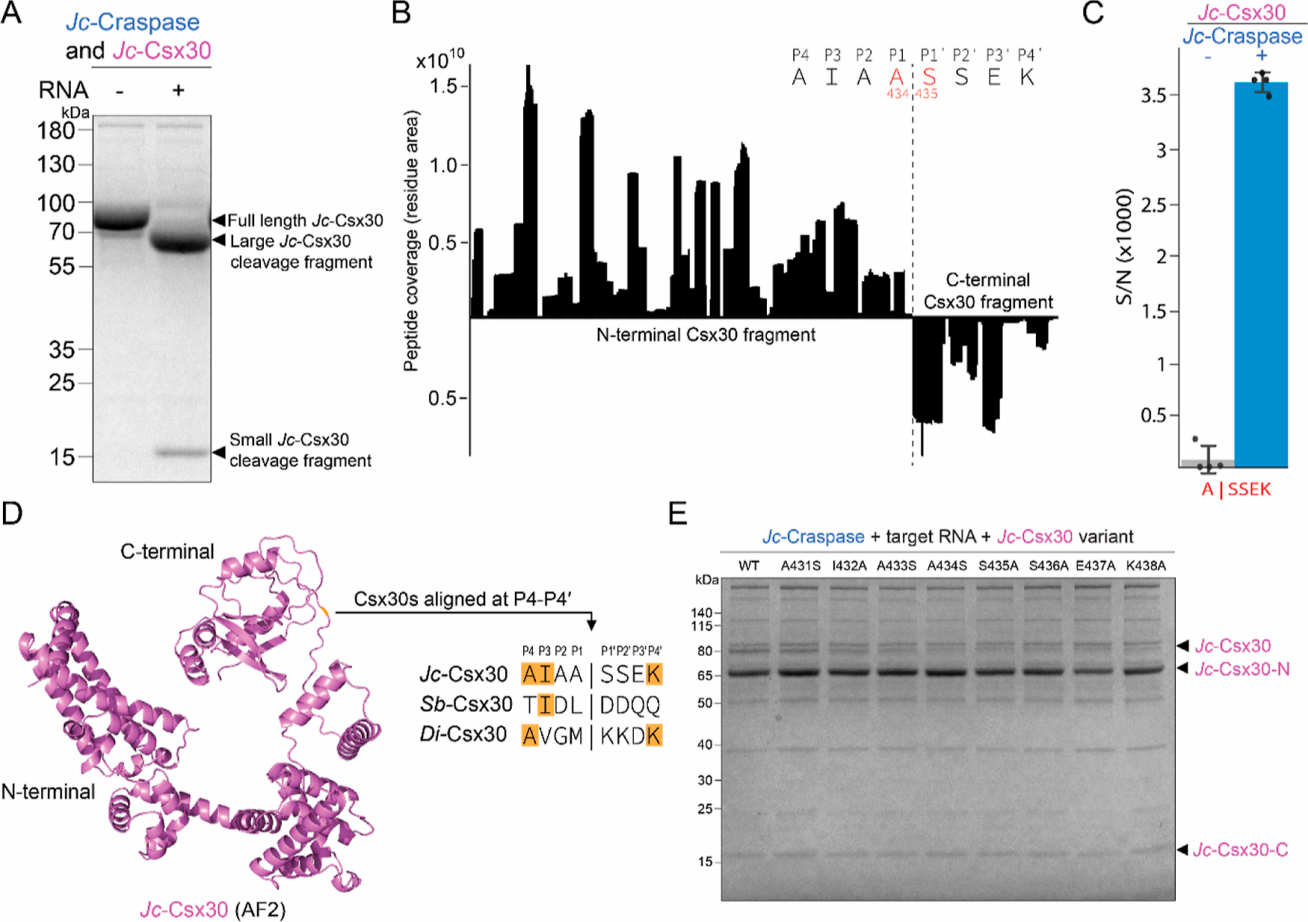
Craspase from *Candidatus* “Jettenia caeni” cleaves Csx30 at A|S. (A) Protein gel after *Jc*-Craspase incubation with *Jc*-Csx30 in the presence or absence of a target RNA. (B) Peptides identified by MS in the large and small *Jc*-Csx30 cleavage fragments, mapped onto the full *Jc*-Csx30 sequence to reveal the cleavage site at A|S. The eight amino acids residues surrounding the cleavage site (P4–P4′) are shown. (C) MS detection of peptides containing the cleavage site A|S in *E. coli* lysates enriched with *Jc*-Csx30, in the absence and presence of *Jc*-Craspase bound to target RNA. S/N indicates signal-to-noise ratios, as determined by TMTpro reporter ion quantification. (D) AlphaFold2^15^ (AF2) model of *Jc*-Csx30 with the position of the cleavage site indicated in orange. Csx30 orthologs are aligned at the cleavage site. Matching residues are indicated in orange. (E) Protein gel after incubation of *Jc*-Craspase with target RNA and *Jc*-Csx30 variants containing individual amino acid substitutions around the cleavage site. The large N-terminal and the small C-terminal cleavage fragments are indicated as *Jc*-Csx30-N and *Jc*-Csx30-C, respectively.

Substrate recognition in the physical context of the target protein is reminiscent of gasdermin cleavage by certain eukaryotic caspases.^11^ It is believed that the high-affinity interaction between caspase and gasdermin provides priming interactions for the insertion of the targeted protein region into the catalytic pocket, bypassing the requirement for a specific motif in the cleavage site. In Craspase, the C-terminal of Csx30 was pinpointed to be the primary protein component for successful Craspase interaction and cleavage.^4^ Analysis of the electrostatic surfaces of the TPR-CHAT proteolytic pocket at the Csx30 binding site revealed a large variety in charge distribution between orthologs (Figure 2A), most likely presenting the molecular basis for differential Csx30 recognition. Indeed, we did not observe cleavage of *Jc*-Craspase on *Sb*-Csx30 nor did we see cleavage of *Sb*-Craspase on *Jc*-Csx30 (Figure 2B), indicating that naturally occurring combinations of Craspase and Csx30 evolved to be specific for each other. The charge complementarity between the CHAT active pocket and the linker of its native Csx30 substrate (Figure 2A) likely contributes to the absence of cross-reactivity. Analysis of Craspase structures in interaction with Csx30 should allow further detailing of the structural recognition code.

**Figure 2:**
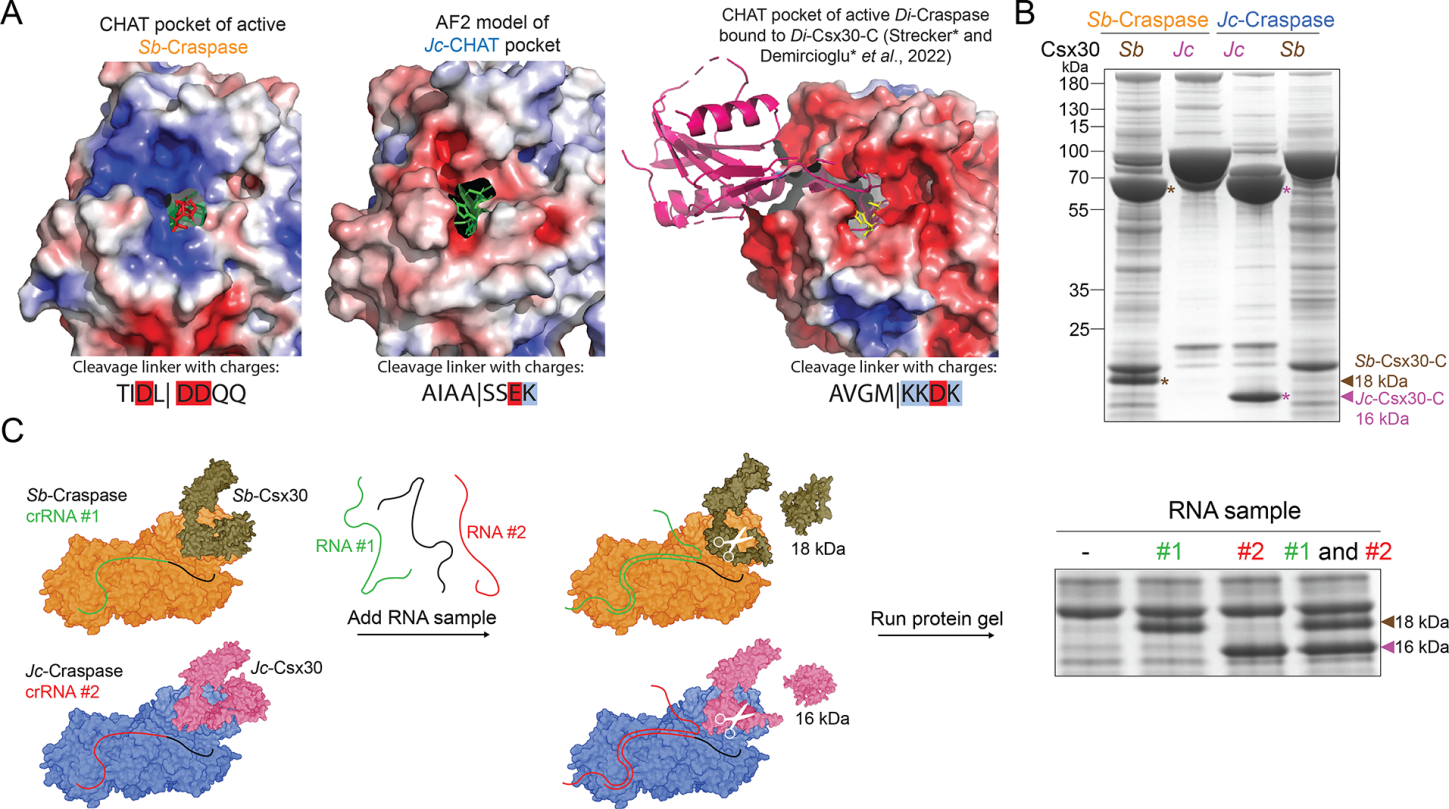
Proteolytic activity of Craspase orthologs is specific toward the corresponding Csx30 target protein. (A) Electrostatic surface maps of the CHAT pockets from TPR-CHAT orthologs and the charges in the cleavage sequence of the corresponding Csx30. Red indicates a negative charge, blue indicates a positive charge, and white indicates a neutral charge. Proteolytic residues are represented as sticks in red (*Sb*-TPR-CHAT), green (*Jc*-TPR-CHAT), and yellow (*Di*-TPR-CHAT). *Di*-Csx30-C is in pink. (B) Protein gel after incubation of *Jc*-Craspase and *Sb*-Craspase with either *Jc*-Csx30 or *Sb*-Csx30 in the presence of target RNA. Cleavage products of *Sb*-Csx30 and *Jc*-Csx30 are indicated with brown and pink asterisks, respectively. (C) Schematic of the multiplexed RNA detection assay in which *Sb*-Craspase and *Jc*-Craspase are programed with specific crRNAs to recognize target RNAs of choice. Upon addition of an RNA sample, each ortholog responds to a different RNA variant to cleave the respective Csx30 protein. This yields C-terminal Csx30 fragments with different sizes (18 kDa for *Sb*-Csx30 and 16 kDa for *Jc*-Csx30). The presence or absence of bands at 18 and 16 kDa on a protein gel reveals whether the target RNAs were in the RNA sample.

We sought to exploit the specificity of Craspase orthologs by creation of a multiplexed Craspase-based assay for detecting and discerning multiple RNA variants in the same reaction. In this assay, the Craspase orthologs are each programed with a specific crRNA to recognize different RNA species and are combined with their native Csx30 proteins. Upon incubation with an RNA sample, the pattern of the resulting Csx30 C-terminal fragments (16 and 18 kDa for cleaved *Jc*-Csx30 and *Sb*-Csx30, respectively) enables deduction of which Craspase was activated and consequently which RNAs were present in the sample (Figure 2C). We could successfully detect and discern either one RNA variant or both variants in a single reaction, demonstrating multiplexed RNA detection (Figure 2C). To circumvent the need for a protein gel readout, a recent study fused a fluorescent protein to the C-terminal side of Csx30.^12^ This allows measurement of the time-dependent increase in fluorescence intensity upon Csx30 cleavage, allowing picomolar detection sensitivity.

In conclusion, we highlight that although Csx30 cleavage by RNA-activated Craspase is conserved across species, Craspase orthologs do not recognize a consensus motif. This provides distinct benefits for biotechnological applications that require multiplexed cleavage specificities, for which we provide a proof-of-principle in this study. By programing the Craspases to recognize specific target RNA of pathogens, such as COVID-19 variants,^12^ one could exploit this assay for point-of-care diagnostics. This is similar to the Cas13- and Cas12-based multiplexing assays, where multiple variants can be detected in one-pot reactions using effector orthologs and different RNA or DNA reporters.^13,14^ An advantage of the Craspase multiplexing assay is its use of protein-based reporters, which may be useful when reporter stability is desired. The assay could in principle be expanded to detection of more RNA variants by adding in additional appropriately programed Craspase orthologs, provided that all are specific toward their own Csx30. So, besides precise and controllable protease action, Craspase possesses yet another unique feature: a nonconserved cleavage site across protease orthologs. This, in combination with its structural recognition requirements, renders Craspase a protease with high selectivity for Csx30 and potentially a narrow range of additional substrates.

## Materials and Methods

### Plasmid Generation

Plasmid generation and transformation were performed according to earlier described protocols.^1^ Used primers and ordered DNA sequences are listed in Tables S1 and S2, respectively. To construct pJc-GRAMP-CRISPR, a coding sequence corresponding to an *E. coli* codon-optimized *Jc*-gRAMP protein variant was cloned downstream of an N-terminal Twin-Strep-Tag II, SUMO-tag, and the LacI repressed T7 promoter on the plasmid 13S-S (encoding spectinomycin resistance for selection) (Berkeley MacroLab). For the crRNA part, a CRISPR array starting with a LacI repressed T7 promoter and the native *Candidatus* “Jettenia caeni” leader sequence, followed by six native repeats interspaced by five times the twelfth spacer in the native CRISPR array (5′-CAAGGACGTTGGGAGAAACCAGTATATCAATCGCAAG) was cloned in the same plasmid. To construct pJc-TPR-CHAT, a coding sequence corresponding to an *E. coli* codon-optimized Jc-TPR-CHAT protein variant was cloned downstream of the LacI repressed T7 promoter on the plasmid pACYC Duet-1. To construct pJc-Csx30, a coding sequence corresponding to an *E. coli* codon-optimized Jc-Csx30 protein variant was cloned to replace Sb-Csx30 on pTag-Csx30.^1^

### Protein Purification and Visualization

Protein purification [from 8 L of *E. coli* BL21(AI) cells containing *Jc*-Craspase or *Jc*-Csx30 variant] and visualization on protein gels were performed according to an earlier described protocol.^1^ SurePAGE (10 × 8) (GenScript) gels were used in MOPS buffer at 160 V for 45–60 min.

### *Jc-*Csx30 Cleavage Assays

*Jc*-Csx30 cleavage reactions were performed in 10 μL reaction volume, containing 5 μL of 5 ng/μL *Jc*-Craspase, 5 μL of 6 ng/μL *Jc*-Csx30 protein, 2 μL of 50 μM in vitro generated target RNA, 100 mM Tris, 150 mM NaCl, and 10 mM DTT. Reactions were run for 1 h at 37 °C.

### Cleavage Position Determination

In Figure 1B, MS was performed according to an earlier described protocol.^1^ Protein digestions were performed by using chymotrypsin. Detected peptides were mapped onto the *Jc*-Csx30 amino acid sequence and are shown in Tables S3 and S4.

In Figure 1C, *Jc*-Csx30 (20 pmol) was incubated overnight (18 h, 30 °C, 350 rpm) with or without *Jc*-Craspase (0.5 pmol) and its target RNA (10 pmol) in four biological replicates. The solution consisted of 25 μL with a final concentration of 100 mM 4-(2-hydroxyethyl)-1-piperazine ethanesulfonic acid (HEPES), 100 mM NaCl, 10 mM dithiothreitol (DTT), and 5 mM ethylenediaminetetraacetic acid (EDTA) in Milli-Q water.

The digestion was stopped with an inactivation mix for a final volume of 100 μL, containing 2.5 M GuHCl, 250 mM HEPES, 10 mM tris(2-carboxyethyl)phosphine (TCEP), and 40 mM 2-chroloacetamide (CAA). *E. coli* lysates (cells lysed under native conditions with a hypotonic buffer and probe sonication) were also added to the samples for a total amount of 50 μg. The mix was incubated at 95 °C and 600 rpm for 10 min to denature proteins and reduce-alkylate cysteine residues. The individual replicates were labeled with 200 μg of TMTpro reagents, incubated for 60 min at RT. The labeling reaction was quenched with 100 mM ammonium bicarbonate (AMBIC), incubated for 30 min at RT. Individual samples were pooled and SP3 cleanup^16^ was performed to remove excess reagents, with the beads finally resuspended in 100 mM HEPES for a final concentration of 1 μg/μL. Protein digestion was performed with a 1:50 trypsin/protein ratio overnight (20 h, 37 °C, 350 rpm). Beads were sonicated, and the supernatant was transferred to a new tube, with 10% of the digest removed as the nonenriched sample. Tryptic peptides in the enriched fraction were tagged with undecanal at a ratio of 1:50 protein/undecanal and 50 mM sodium cyanoborohydride (NaBH_3_CN) in 40% ethanol (EtOH) and incubated for 90 min at 50 °C, 450 rpm. Tryptic peptides were depleted by loading the solution to a conditioned SepPak (Waters, 50 mg capacity) desalting column and retrieving the flow-through. An estimated 500 ng of both nonenriched and N-termini enriched samples were acidified and loaded on EvoTip Pure trap columns with the low input protocol and queued for MS analysis.

Samples were measured with an EvoSep One liquid chromatography system in line with an Orbitrap Eclipse trybrid mass spectrometer, equipped with a FAIMSpro ion mobility device. Peptide separation was performed with the 20SPD 58 min gradient method using an Aurora Elite TS Generation 3, 15 cm column (IonOpticks). Peptides were injected with nanospray ionization in the positive ion mode with a spray voltage of 2300 V, an ion transfer tube temperature of 240 °C, and a carrier gas flow of 3.6 L/min. The instrument was operated in the data-dependent acquisition mode, and two experiments utilizing different compensation voltages were used during measurement (−45 and −65 V) with otherwise identical settings. MS scans were acquired in the Orbitrap at a 60,000 resolution, with a scan range of 375–1500, maximum injection time (IT) at 118 ms, normalized AGC target at 300%, and RF lens at 40%. The filters used for precursor selection were: MIPS mode peptide, allowed charged states between 2 and 7, dynamic exclusion after 2 times for 30 s with a 10 ppm tolerance, a minimum intensity threshold at 20,000, and a precursor fit at 70% with a 1.2 *m*/*z* fit window. MS/MS scans were recorded with a total cycle time of 1 s for each compensation voltage. Precursors were isolated in the quadrupole with a 1.2 *m*/*z* isolation window and fragmented with HCD at NCE 34%. Scans were acquired in the Orbitrap in the centroid mode with a resolution of 30,000, maximum IT of 54 ms, and normalized AGC target at 100%.

Raw data were searched with Proteome Discoverer v2.4. The nonenriched and enriched samples were added as fractions, and TMTpro quantification was selected. The Sequest HT engine was used for PSM detection, and Percolator was used for the FDR control (1% strict, 5% relaxed). Craspase and Csx30 sequences were added to the *E. coli* reference proteome (UniprotKB, 4360 sequences, accessed 17/01/2023). The search was performed with ArgC specificity with semispecific N-terminal search, with a peptide length between 6 and 46. Methionine oxidation (+15.995 Da), asparagine deamidation (+0.984), N-terminal acetyl (+42.011), and TMTpro (+304.207) were added as variable modifications, while cysteine carbamidomethylation (+57.021) and lysine TMTpro modification (+304.207) were added as fixed modifications. TMT quantification was performed on unique and razor peptides using the Reporter Ions Quantifier, with normalization on the median of total peptide amount per channel (N-terminal TMTpro-modified peptides excluded). Filters for quantification were set to 10 S/N threshold and 50% coisolation threshold. Detected peptides are shown in the Supplementary Excel File.

### In Vitro Generation of RNA Cognate to the crRNA in *Jc*-Craspase

Target RNA generation was done with a gBlock containing the T7 promoter and RNA complementary to the crRNA in purified *Jc*-Crapase, according to an earlier described protocol.^1^

### Multiplexed Craspase Assay

For the multiplexed Craspase assay, reactions were prepared containing purified proteins (5 μL of 5 ng/μL *Jc*-Craspase, 2.5 μL of 2.5 ng/μL *Sb*-Craspase R294A D698A, 5 μL of 5 ng/μL *Sb*-Csx30, and 5 μL of 6 ng/μL *Jc*-Csx30). Two μL amount of 50 μM in vitro generated RNA cognate to the crRNA in *Jc*-Craspase and/or *Sb*-Craspase was added, and reactions were incubated at 37 °C for 2 h.

## Supporting Information Available

Peptides identified by MS (XLSX)
Used primers, gBlocks, and MS analysis data (PDF)
