# Supplementary material for: Craspase Orthologs Cleave a Nonconserved Site in Target Protein Csx30
Source: ACS Chem Biol. 2024 Apr 11;19(5):1051–5. doi: 10.1021/acschembio.3c00788 (PMC11106740; doi:10.1021/acschembio.3c00788)
Supplement: Supplementary file 2 — cb3c00788_si_002.pdf [file cb3c00788_si_002.pdf]

Supporting Information

Craspase orthologs cleave a non-conserved site in target protein Csx30

Sam P. B. van Beljouw[1,2], Anna C. Haagsma[1,2], Konstantinos Kalogeropoulos[3], Martin Pabst[4] and Stan J. J. Brouns*[1,2]

[1]Department of Bionanoscience, Delft University of Technology, 2629 HZ, Delft, Netherlands. [2]Kavli Institute of Nanoscience, 2629 HZ, Delft, Netherlands. [3]Department of Biotechnology and Biomedicine, 2800 Kgs. Lyngby, Denmark. [4]Department of Biotechnology, Delft University of Technology, 2629 HZ Delft, Netherlands.

Email address: stanbrouns@gmail.com

Table S1. Primers used in this study.

Sequence (5′→3′)	Description (Forward/Reverse couples)
GATCGAGGAAAACCTGTACTTCCAATCCAATGCAATGCATACCATTCTGCCGATTC	On *Jc*-gRAMP gBlock for pJc-GRAMP-CRISPR construction
GATCGCGGATCCGTTATCCACTTCCAATGTTATTATTATTTGGCGATTTCAATATCTT C	On *Jc*-gRAMP gBlock for pJc-GRAMP-CRISPR construction
TAATAACATTGGAAGTGGATAACGGATCCGCGATC	On 13S-S for pJc-GRAMP-CRISPR construction
TGCATTGGATTGGAAGTACAGGTTTTCCTCGATC	On 13S-S for pJc-GRAMP-CRISPR construction
TGTACAATACGATTATTTACCGGTTTATTGACTACCGGAAG	On 13S-S for Jc-CRISPR insertion
TTTTGCTGAAACCTCCCAGCAATAGACATAAGCGGC	On 13S-S for Jc-CRISPR insertion
ATCCGTAAGCTGCATTGCATTGGATTGGAAGTACAGG	On pTag-Csx30 to construct pJc-Csx30
ATCTCTTTGGAGTGATAATCCTGAAGCCCTAGCATAACC	On pTag-Csx30 to construct pJc-Csx30
TTTCTACAGGGGAATTGTTATCC	On pACYC Duet-1 to construct pJc-TPR-CHAT
TACTAGCGCAGCTTAATTAACC	On pACYC Duet-1 to construct pJc-TPR-CHAT

Table S2. gBlocks used in this study.

gBlock	Sequence 5’-3’
To generate target RNA for *Jc*-Craspase	TCGATCAGAGCGCTCTTACGTAATACGACTCACTATAGGTGTACGCTATTCAGGGATTGACTGATACCGGAAGACATCTCTTGCGATT GATATACTGGTTTCTCCCAACGTCCTTGTGTAGCCTTTTGCGTCTTAAGCCCAATGAAAAACCTATGCACGA
Codon-optimized *Jc*-gRAMP	ATGCATACCATTCTGCCGATTCATCTGACCTTTCTGGAACCGTATCGTCTGGCCGAATGGCATGCAAAAGCAGATCGCAAAAAAAACA AACGTTATCTGCGTGGTATGAGCTTTGCACAGTGGCATAAAGATAAAGATGGTATCGGCAAACCGTATATTACCGGTACACTGCTGC GTAGCGCAGTTCTGAATGCAGCAGAAGAACTGATTAGCCTGAATCAAGGTATGTGGGCAAAAGAACCGTGTTGCAATGGTAAATTTG AAACCGAGAAAGATAAACCGGCAGTTCTGCGTAAACGTCCGACCATTCAGTGGAAAACCGGTCGTCCGGCAATTTGTGATCCGGAAA AACAAGAAAAAAAAGATGCATGTCCGCTGTGTATGCTGCTGGGTCGTTTTGATAAAGCAGGTAAACGTCATCGTGACAACAAATATG ATAAACACGATTACGACATCCACTTTGACAACCTGAATCTGATCACCGACAAAAAATTCAGCCATCCTGATGATATTGCCAGCGAACG TATTCTGAATCGTGTTGATTATACCACCGGTAAAGCCCACGATTATTTCAAAGTTTGGGAAGTCGATGATGATCAGTGGTGGCAGTTT ACCGGCACCATTACCATGCATGATGATTGTAGCAAAGCAAAAGGTCTGCTGCTGGCAAGCCTGTGTTTTGTTGATAAACTGTGTGGTG CACTGTGCCGTATTGAAGTTACCGGCAATAATAGCCAGGATGAGAATAAAGAATATGCCCATCCGGATACCGGCATTATTACCAGCCT GAACCTGAAATATCAGAACAACAGCACCATTCACCAGGATGCAGTTCCGCTGAGCGGTAGCGCACATGATAATGATGAACCGCCTGT TCATGATAACGATAGCAGCCTGGATAATGATACAATTACCCTGCTGAGCATGAAGGCCAAAGAAATTGTTGGTGCATTTCGTGAAAG CGGCAAAATTGAAAAAGCACGTACCCTGGCAGATGTTATTCGTGCAATGCGTCTGCAGAAACCGGATATTTGGGAAAAACTGCCGAA AGGCATTAACGATAAACATCATCTGTGGGATCGTGAAGTGAATGGTAAAAAACTGCGCAACATTCTGGAAGAACTGTGGCGTCTGAT GAATAAACGTAATGCATGGCGTACCTTTTGTGAAGTTCTGGGTAATGAACTGTATCGCTGCTACAAAGAAAAAACCGGTGGCATTGTT

	CTGCGTTTTCGCACCCTGGGTGAAACCGAATATTATCCTGAACCGGAAAAAACGGAACCGTGTCTGATTAGCGATAATAGCATTCCGA<br>TTACACCGCTTGGTGGTGTTAAAGAATGGATTATTATCGGTCGTCTGAAAGCAGAAACCCCGTTTTATTTCGGTGTTCAGAGCAGCTT<br>TGATAGCACCCAGGATGATCTGGATCTGGTTCCGGATATTGTTAACACCGATGAAAAACTGGAAGCCAATGAACAGACCAGCTTTCG<br>CATTCTGATGGATAAAAAAGGTCGTTATCGTATTCCGCGTAGCCTGATTCGTGGTGTGCTGCGTCGTGATCTGCGTACCGCATTTGGT<br>GGTAGCGGTTGTATTGTTGAACTGGGTCGTATGATTCCGTGTGATTGTAAAGTTTGTGCCATCATGCGTAAAATCACCGTTATGGATA<br>GCCGCAGCGAAAACATTGAACTGCCTGATATTCGCTATCGCATTCGTCTGAATCCGTATACCGCAACCGTTGATGAAGGTGCCCTGTT<br>TGATATGGAAATTGGTCCGGAAGGTATTACCTTTCCGTTTGTGTTTCGTTATCGCGGTGAAGATGCACTGCCTCGTGAACTGTGGTCA<br>GTTATTCGTTATTGGATGGATGGTATGGCATGGTTAGGTGGTAGTGGTAGCACCGGCAAAGGTCGCTTTGCACTGATTGATATTAAA<br>GTGTTTGAATGGGATCTGTGCAATGAAGAAGGCCTGAAAGCATATATTTGTAGCCGTGGTCTGCGTGGCATCGAAAAAGAAGTTCTG<br>CTGGAAAACAAAACCATTGCGGAAATTACCAACCTGTTCAAAACCGAAGAAGTCAAATTCTTCGAGAGCTACAGCAAACACATTAAA<br>CAGCTGTGTCATGAGTGCATCATTAACCAGATTAGCTTTCTGTGGGGTCTGCGTAGCTATTATGAATATCTGGGTCCTCTGTGGACCG<br>AAGTGAAATATGAAATCAAAATTGCAAGTCCGCTGCTGAGTAGCGATACCATTAGCGCACTGCTGAACAAAGATAACATTGATTGCA<br>TTGCCTACGAGAAACGCAAATGGGAAAATGGTGGTATCAAATTTGTGCCGACCATTAAAGGTGAAACCATTCGTGGTATTGTTCGTAT<br>GGCAGTTGGTAAACGTAGCGGTGATTTAGGTATGGATGATCATGAAGATTGCAGCTGTACCCTGTGTACCATTTTTGGCAATGAACA<br>TGAAGCAGGCAAACTGCGTTTTGAAGATCTGGAAGTTGTTGAAGAGAAACTGCCGTCAGAACAGAATAGCGATAGCAACAAAATTC<br>CGTTTGGTCCTGTGCAGGATGGTGATGGTAATCGTGAAAAAGAATGTGTTACCGCAGTGAAAAGCTATAAAAAGAAACTGATTGACC<br>ACGTGGCCATCGATCGTTTTCATGGTGGTGCCGAAGATAAAATGAAATTTAACACCCTGCCGCTGGCAGGTAGCTTTGAAAAACCGA<br>TTATTCTGAAAGGCCGTTTTTGGATCAAAAAAGACATCGTGAAAGACTATAAGAAAAAGATCGAAGATGCCATGGTGGATATTCGTG<br>ATGGTCTGTATCCGATTGGTGGTAAAACCGGCATTGGTTATGGTTGGGTTACCGATCTGACCATCCTGAATCCGCAGAGCGGTTTTCA<br>GATTCCGGTTAAAAAAGATATTTCACCGGAACCGGGTACGTATAGCACCTATCCGAGCCATAGCACCCCGAGTCTGAATAAAGGTCA<br>TATCTATTATCCGCATTACTTTCTGGCTCCGGCAAATACCGTTCACCGTGAACAAGAAATGATTGGCCATGAACAGTTTCATAAAGAAC<br>AGAAAGGTGAACTGCTGGTGAGCGGTAAAATTGTTTGTACCCTGAAAACAGTGACACCGCTGATTATTCCGGATACGGAAAATGAAG<br>ATGCCTTTGGCCTGCAGAATACCTATAGCGGTCATAAAAACTACCAGTTCTTTCACATCAACGATGAGATTATGGTTCCGGGTAGCGA<br>AATTCGCGGTATGATTAGCAGCGTTTATGAAGCAATTACCAATAGCTGCTTTCGCGTTTACGATGAAACCAAATATATCACCCGTCGTC<br>TGAGCCCTGAAAAAAAGGATGAAAGCAACGACAAAAACAAAAGCCAGGACGATGCAAGCCAGAAAATTCGTAAAGGTCTGGTGAA<br>AAAAACCGACGAAGGCTTTAGCATTATTGAGGTTGAACGCTATAGCATGAAAACCAAAGGTGGCACCAAACTGGTTGATAAAGTTTA<br>TCGTCTGCCGCTGTATGATAGCGAAGCAGTTATTGCAAGCATTCAGTTTGAACAGTATGGCGAGAAAAACGAAAAACGCAATGCAAA<br>AATTCGCGCAGCCATTAAACGTAACGAAGTTATTGCCGAAGTTGCCCGTAAAAATCTGATTTTCCTGCGTAGTCTGACACCTGAGGAA<br>CTGAAAAAAGTGCTGCAGGGTGAAATTCTGGTCAAGTTTAGTCTGAAAAGTGGCAAAAACCCGAATGATTATCTGGCTGAACTGCAT<br>GAAAATGGCACCGAACGTGGTCTGATTAAATTCACCGGTCTGAATATGGTGAACATCAAAAATGTGAACGAAGAGGACAAAGACTTC<br>AACGATACCTGGGATTGGGAGAAACTGAACATTTTTCATAACGCCCATGAGAAACGTAACAGCCTGAAACAGGGTTATCCGCGTCCG<br>GTTCTGAAATTTATCAAAGATCGTGTGGAATACACCATTCCGAAACGTTGTGAACGTATTTTTTGTATCCCGGTCAAAAACACCATCGA<br>GTATAAAGTTAGCAGCAAAGTGTGCAAACAGTACAAAGATGTTCTGAGCGACTATGAAAAGAACTTTGGCCATATCAACAAGATCTT<br>CACCACCAAAATTCAGAAACGCGAACTGACCGATGGTGATCTGGTTTATTTCATTCCGAACGAAGGTGCAGATAAAACCGTTCAGGC<br>AATTATGCCGGTGCCGCTGAGCCGTATTACCGATAGCCGTACACTGGGTGAACGCCTGCCGCATAAAAACCTGCTGCCGTGTGTTCAT<br>GAAGTTAATGAAGGCCTGCTGAGTGGTATTCTGGATAGCCTGGACAAAAAACTTCTGAGCATTCATCCGGAAGGCCTGTGTCCGACC<br>TGTCGTCTGTTTGGCACCACCTATTACAAAGGTCGTGTTCGTTTTGGTTTTGCCAACCTGATGAACAAACCGAAATGGCTGACCGAAC<br>GCGAAAATGGTTGTGGTGGTTATGTTACACTGCCGCTGCTGGAACGTCCGCGTCTGACCTGGTCAGTTCCGAGCGATAAATGTGATG<br>TTCCGGGTCGTAAATTCTACATTCATCATAATGGTTGGCAAGAGGTGCTGCGCAATAATGATATTACCCCGAAAACCGAAAATAACCG<br>TACCGTTGAACCTCTGGCAGCCGATAATCGTTTTACCTTTGATGTGTATTTTGAGAATCTGCGTGAATGGGAACTGGGCCTGCTGTGTT<br>ATTGTCTGGAACTGGAACCTGGTATGGGTCATAAATTAGGCATGGGTAAACCGATGGGTTTTGGTAGCGTGAAAATTGCCATTGAAC<br>GTCTGCAAACCTTTACCGTTCATCAAGATGGCATTAATTGGAAACCGTCCGAAAACGAAATTGGTGTGTATGTTCAGAAAGGACGTG<br>AGAAACTGGTGGAATGGTTTACCCCGAGCGCACCGCACAAAAACATGGAATGGAATGGCGTGAAACATATTAAAGATCTGCGTTCCT<br>TACTGTCCATTCCGGGTGATAAACCGACCGTTAAATATCCGACACTGAATAAGGATGCAGAAGGTGCAATTAGCGATTATACCTATGA<br>GCGTCTGTCAGATACCAAACTGCTGCCGCATGATAAACGCGTGGAATATCTGCGCACCCCGTGGTCACCGTGGAATGCATTTGTAAA<br>AGAAGCAGAATATAGCCCGAGCGAAAAATCGGATGAAAAAGGACGCGAAACAATTCGCACCAAACCTAAAAGCCTGCCGAGCGTTA<br>AAAGCATTGGTAAAGTTAAATGGTTCGATGAAGGCAAAGGCTTTGGTATCCTGATCATGGATGATGGCAAAGAAGTTAGCATTAGCA<br>AAAATAGCATCCGTGGTAACATCCTGCTGAAAAAGGGTCAGAAAGTGACCTTTCATATTGTGCAGGGTCTGATTCCGAAAGCGGAAG<br>ATATTGAAATCGCCAAATAA
Codon-optimized *Jc*-TPR-CHAT	ATTGTGAGCGGATAACAATTCCCCTGTAGAAATAATTTTGTTTAACTTTAATAAGGAGATATACCATGAAAAACCGCGTGCAGATTGA<br>AGCCATTATTCGTAATCTGCAGGGTGCAGCACGTGATAGCAAAACCAATAAACTGAGCGAAAACATTATCGCCTATGACGAGTATCG<br>CAAAATTCATAAAAGCGCAAGCCTGTATCAGTTTGGTATTATTCCGGCAAAAGAAAGCAGCAGCGTTCTGGCAGAAAATGAAACCAA<br>TCATGTTGCATGCGAAAACGCCATTTTTGAAATGGCCGAAAAGAACATCGAAAACTTTAGCAGCGAAGATATCCACAAGAAACGCAA<br>AGAAACCATTGAAAGCGCACTGCGTCTGCTGATGGGTCTGTATAAAGATCGTCACGAAAAACTGCAGCCTCGTACCTTTGTTCTGATT<br>GCAAAAGCATATCTGCTGCGTAGCCTGATTACCCGTCCGAAAGGTATTACCATTCCTGAGAAAAAGAAAGAAGCCCTGAAGAAAGGC<br>ATTGGCTTTGTGGAAAGCGCCATTAAAAAGATTCAGAGCAGTGAAAACATTCTGAGCCATAGCAGCGATATTGATCTGCTGGAAAAA<br>GCATGGCGTATTAAAAGCCAGCTGTATCTGGAATATTACCGCGTGAATAAAGATGAGTGCGATAAGAATACCCTGAAAGAGGTTCTG<br>GAAAACAGCCTGATTTCAGGTTGCGATAAATTCGACAAAAACATCGAAGATGTGCAGATCGCCATTCGTTATTGTGAACTGGAAAGC<br>AGCCGTGAATATCTGGAACAAATTATCAGCAGCCATCTGGAAGGCATCGAATTTGAAAAAGCACGTGCATATAAACTGCTGGAACTT<br>GAAAACGAAAACGAAGATGAAATTCGCAAGAGCATGAAAGTGGTGATCGAAGAATATCTGAGCGGTTTTAGCGATCCGCTGTGGGA<br>AGATGCAGTTGAATTTATCAACAAACTGAAAAGCGATAACAAAAATTGCTGGAAAGAACTGAGCCTGGACATGTATAAAGTTTGCCG<br>TGAACAAGAAGCAGAAACCGCCAGCCTGCATCTGCGTTGGTATTGGAGCCGTCAGCGTCGTCTGTATGATCTGGCATTTATTGCAGC<br>CGATAAAGAAGAAGAGAAAGCCAAAATTGCAGACAGCCTGAAAAGCCGTCTGAGCCTGCGTTGGAGCGCACTGGAAGAAACCGGT

	AAGAAAAGCAAAAACAAACGCGAGAAAGAAGAAATTAGCCGTATTCTGGAAGCCGAAGCAGTTGCAATGTTAGGTGGTTATATCAA<br>AGGTGCCCGTAAAATTCTGAAGAAACGTCGTCGTCCGCTGCCGGATGAACAGCGTAGCATTCCGAAAGATTGGATTGTGATCCACTT<br>TTATGTGAACCAGCTGGAAAATAAGTGCTATGCCCTGATCTATAACAAAGATGAGAATACCTGGAAATGCGAATTTGTGAAAGAATA<br>TCAGCGCCTGTTTCATGTTTTTCTGACCTGGCAGACCAATTATAACCGTTGTAAAGAACGTGCCGCAGATAGCCTGGTTCAGCTGTGT<br>AAAGAAATTGGTAATGCAATGCCGTTTCTGTTCGATGAATGCATTATTCCGCAGGATAAAAACGTGCTGTTTATCCCGCATGATTTTCT<br>GCATCGTCTGCCGCTGCATGGTGCAATTCATGAAAAGAATAATGGTGTGTTCCTGGAAAATCATCCGTGTTGTTATCTGCCTGCATGG<br>TCATTTGCAGCCAAAGAAAATAATGCAGTTGTGCAGGGTAGCATCCTGCTGAAAAACTTTCCGGAATACAGCTATGAAGAACTGGTT<br>AGCAATAGCACCCTGTGGACCAGTCCGGTTAAAGATCCGGCAAGTCCGGATGATCTGAAAACCATTATTGCATCACCGGAAATGCTG<br>GTTATTCTGTGTCATGGTGAAGCAGATGCCGTTAATCCGTTTAATGCCCGTCTGAAACTGACCGGTAATGGTATTAGTCATCTGGAAA<br>TTCTGCAGAGCACCAAAATGATTCTGAAAGGCAGCAAAATTATCCTGGGTGCATGTGAAACCGATCTGGTTCCGCCTCTGAGCGATAT<br>TATGGATGAACATCTGAGCATTACCACCGCATTTCTGACCAATGATGCACGTGAAATTCTGGGCACCATGTATGAAGCACTGGATGTT<br>CGTATTAGCAGCATCATCCAGAAAATCTATCGCCAAGAACATTACAGCAGCATGATGAAACAGCTGTGGGAGTGGCAGAAAGTTGGT<br>GTTGAAAATTATCGCGAAAATGGTGATACACCGGCATTCTATAACACCGTTGTGTTTCGTGTTATTGGCCTGAGCATTTAATCTACTAG<br>CGCAGCTTAATTAACCTAGGCTGCTGCCACCGCTGAGCAATAACTAGC
Codon-optimized *Jc*-Csx30	ATGCAGCTTACGGATAAATCGCGCAACGAATTGTTCTCAGCTTTGTTGGAGTGGGGTAAATCCCATATGTTGTCGCCGGAGATCGTTC<br>AGGACATTAGTGAAATTGAAGAAAACTGTGAACCATTTATCAAGTCAGACGAATTTACTAATTTTCTTTTAAATAGTGTAGAGAAAAT<br>TCGTCGCCAGGTAGAATTTGCAATTTCGCTTTTCACCAACATCGAGTTGGTCACTGCAACCGATGAGGATTCATTATGGCACGACGCT<br>GAGGTGAGCTTTATTGCATTCGATCGTGTTAACTACTTAATGGAGGTGCTTCAATTCTTAATTGTACCGCGCAAAAAGGAAATCGCTG<br>AAAAGGCAATGAAGGCATTGGACACCATTTTTGAGAAAACAATTGGTTGGCTTGAAGAGTCAGAGTTCTCCCCGCTTCGTCTGGTCG<br>TATTGAACGAAAGTCGCCGCTACCACCTTGAACAGATTCCAGAGGACGAGCGTTATCGTTTTCCCTGGTATGAATTGTACTCAGACTA<br>CTCTGAAAATACATTAGAGATCATCATCGAGAATTTCGACACATTCCTTTCCGGCAAATGGGAGAAGTTGACCCGCGAAATCCCTCAA<br>GAGTACCTGCACGAAATTAGCTTAGAGTTAAAACGTGACAAATTACTGTTGTCACGTATTAAACAAGAGGCCTCATATCACAAGCGTT<br>TGTTAGCAGCCGTAAGTAAGCCAAGTTCATTGAAGTTATGGCGTCTTGGAGACGAGGCGGCTCTTGACTACTTTCTTCCTGGCGGGGT<br>CAAAAAGTCAGGGGCGGTACGCGTTTCTTTGAAATTAATCGAGGATGCGAAGATTGCATTTGAGGCGGATGAAATCTGTTGGAAGTT<br>TCTGGCCGCTTTCTGCGGTCCGAACTTAGATGACAAGCAGCGCCTTTCTTTACTTGACAAGGTGGAGGAGCGCATTAAGAAGATTGAT<br>ATTCATAAAATTAGCGAGAACGAGAATATCTTGAAAACATTAAAATCCTGGTTCGAGGGTAATTGCGACAATGCCAAACTGATTAAA<br>ATTTCGTTTGACACGTGGATCAAGATGATGGAGGAAAAAGCTATCAACATGCATGCAGTAGAATTCGATGAGTCCCCAGAAAAATTA<br>TGGAACGCCATTATGGAACTTCAGAAGGGTCGCATGGGTGCCTCAAACTCCGTTGAAAAGTACATTAATGACTTTGTGAATGGGTGC<br>CGTCAAGTATGGCAGATTTTTAAATCAACCGTAGATGAACCATCCGTGCTTTATGGTGCTCGTGCGATTGCTGCCTCTAGCGAAAAGA<br>GTGTGCAGCCCCGTAAGCTTGAATTGAACAACAACCCGATTTTATTGTCCTTAAAACCCAATCCAAAGGGTGAGTACATCATTCTGAG<br>CTCGCTGGCATTAAAGCGCGTTGTTTTGGGGGAAGGGGTGGAGGACTACGAAAAGATCTGGAATTATTTGGAGCATACAAAAAATG<br>ACTACTGGTGCGGCTGTTTTATTACAAATGACGATAAACCAGACGTTGCATCTGTCCAGCAAATTGAAAATCGTATCTTAGCTAAAAA<br>GACCCGTGACTACAAAAAAGCAATCATCGGAGTCTCACCCGAGAAAATCGTGCTTGAGGAATTTATCCAGGAGCTGCCAGCCGTTAT<br>CTTTGAAGGCAAAGGGCCATTGAAAGATTCGTTGGTAAAAAAAGTAATCATTTTAGTCATCTCTTTGGAGTGA
Jc-CRISPR	TAATCGTATTGTACACGGCCGCATAATCGAAATTAATACGACTCACTATAGGGGAATTGTGAGCGGATAACAATTCCCCATCTTAGTA<br>TATTAGTTAAGTATAAGAAGGAGATATACATATGTACAACACGGCACCCTTCCTGCCAGTATATAACCTCAATGTTTGAGAGGTCTTG<br>AAGACTAAAGGAAGGAATTAATGTCACGGTACCAAGGACGTTGGGAGAAACCAGTATATCAATCGCAAGCTTGAAGACTAAAGGAA<br>GGAATTAATGTCACGGTACCAAGGACGTTGGGAGAAACCAGTATATCAATCGCAAGCTTGAAGACTAAAGGAAGGAATTAATGTCA<br>CGGTACCAAGGACGTTGGGAGAAACCAGTATATCAATCGCAAGCTTGAAGACTAAAGGAAGGAATTAATGTCACGGTACCAAGGAC<br>GTTGGGAGAAACCAGTATATCAATCGCAAGCTTGAAGACTAAAGGAAGGAATTAATGTCACGGTACCAAGGACGTTGGGAGAAACC<br>AGTATATCAATCGCAAGCTTGAAGACTAAAGGAAGGAATTAATGTCACGGTACCAATTGGATATCGGCCGGCCACGCGATCGCTGAC<br>GTCGGTACCCTCGAGTCTGGTAAAGAAACCGCTGCTGCGAAATTTGAACGCCAGCACATGGACTCGTCTACTAGCGCAGCTTAATTAA<br>CCTAGGCTGCTGCCACCGCTGAGCAATAACTAGCATAACCCCTTGGGGCCTCTAAACGGGTCTTGAGGGGTTTTTTGCTGAAACCTC

Table S3. Mass spectrometry analysis of the chymotrypsin-treated large Csx30 fragment. In order to identify the *Jc*-Craspase cleavage site, no assumption of cleavage site preference was made in peptide sequence mapping.

Peptides	-10 log P	Area
SDSEVNQEAKPEVKPEVKPETH	111.24	8.22E7
GARAIAASSEKSVQPRKL	107.46	2.75E6
SDSEVNQEAKPEVKPEVKPETHINL	103.49	2.46E8
KIDIHKISENENILKTL	103.36	9.69E8

ASMSDSEVNQEAKPEVKPEVKPETH	102.53	7.95E7
SMSDSEVNQEAKPEVKPEVKPETHINL	102.11	4.46E7
HAVEFDESPEKLW	101.78	1.84E9
ASMSDSEVNQEAKPEVKPEVKPETHINL	101.68	1.19E9
IKMMEEKAINMHAVEFDESPEKLW	100.34	6.73E8
LIVPRKKEIAEKAM	99.04	2.99E9
SHPQFEKGSSMASMSDSEVNQEAKPEVKPEVKPETH	98.60	1.98E7
SHPQFEKGGGSGGGSGGGAW	98.35	1.77E9
DYFLPGGVKKSGAVRVSL	98.23	3.49E8
KSWFEGNCDNAKL	98.07	1.33E9
KLIEDAKIAFEADEICW	98.01	1.16E9
LPGGVKKSGAVRVSL	96.60	1.05E9
LIVPRKKEIAEKAMK	95.34	1.09E8
KIDIHKISENENILK	95.20	1.07E8
KALDTIFEKTIGW	93.73	1.34E9
IVPRKKEIAEKAM	93.30	8.06E8
KSWFEGNCDNAK	92.70	7.96E6
IKMMEEKAINMHAVEF	92.50	2.49E8
KTLKSWFEGNCDNAKL	92.23	1.25E8
VPRKKEIAEKAM	92.16	1.05E7
KFLAAFCGPNLDDKQRL	90.32	8.18E6
TLKSWFEGNCDNAKL	90.22	1.22E8
LLSRIKQEASYH	90.22	1.32E9
MHAVEFDESPEKLW	89.82	6.88E7
AAFCGPNLDDKQRL	89.81	1.26E8
PGGVKKSGAVRVSL	89.76	8.43E7
HAVEFDESPEKL	89.35	7.86E7
FQSNAMQLTDKSRNELF	89.27	3.01E8
FLPGGVKKSGAVRVSL	88.99	6.43E9
CGPNLDDKQRLSLL	88.56	1.17E7
KAINMHAVEFDESPEKLW	88.48	7.22E6
PQFEKGGGSGGGSGGGAW	88.43	7.26E6
IVPRKKEIAEKAMK	88.29	1.95E7
LLNSVEKIRRQVEF	87.86	3.6E9

DIIEAHREQIGGIEENLY	86.84	3.38E7
AKRQGKEMDSLRF	86.68	5.71E8
CGPNLDDKQRL	86.58	1.65E9
ELQKGRMGASNSVEKY	86.11	6.21E8
DKVEERIKKIDIHKISENENIL	85.53	1.96E8
IVPRKKEIAEKAMKAL	85.33	1.96E8
IKMMEEKAINM	85.15	1.67E9
SSMASMSDSEVNQEAKPEVKPEVKPETHINL	85.01	5.87E7
LYDGIRIQADQTPEDLDMEDNDIIEAH	84.68	1.27E7
SPEIVQDISEIEENCEPF	84.24	4.66E7
SLLDKVEERIKKIDIH	83.17	5.38E8
SEVNQEAKPEVKPEVKPETH	82.63	2.14E6
MEEKAINMHAVEFDESPEKLW	82.57	2.02E7
LYDGIRIQADQTPEDLDMEDNDIIEAHREQ	82.09	1.94E7
MEEKAINMHAVEF	82.08	1.09E7
DKVEERIKKIDIHKISENENILK	81.98	9.71E6
LIVPRKKEIAEKA	81.97	2.16E8
KSWFEGNCDNAKLI	81.92	8.18E7
IKMMEEKAINMH	81.77	2.4E8
TNIELVTATDEDSLWHD	81.13	7.75E6
KIDIHKISENEN	80.84	1.02E6
KRLLAAVSKPSSL	80.73	4.44E6
RLGDEAALDYFLPGGVKKSGAVRVSL	80.73	1.65E7
LIVPRKKEIAEKAMKAL	80.63	6.22E8
EKLTREIPQEY	80.51	1.53E9
HLEQIPEDERY	80.14	1.53E9
KKEIAEKAMKALDTIF	80.13	2.45E8
NDIIEAHREQIGGIEENLY	80.11	2.8E7
KYINDFVNGCRQVW	79.97	2.48E6
AINMHAVEFDESPEKL	79.92	8.49E5
PEVKPEVKPETHINL	79.80	3.12E7
KIDIHKISENENIL	79.62	3.47E7
FEKGGGSGGGSGGGAW	79.42	3.8E7
PRKKEIAEKAM	79.05	6.1E6

PGGVKKSGAVR	78.68	1.4E7
LKSWFEGNCDNAKL	78.19	1.2E6
AMKALDTIFEK	78.13	4.72E6
LSRIKQEASYH	77.97	5.17E7
ELQKGRMGASNSVE	77.83	1.43E6
DKVEERIKKIDIH	77.71	4.96E8
LPGGVKKSGAVRVS	77.69	3.26E7
SHPQFEKGSSM	77.61	1.07E8
LDKVEERIKKIDIH	77.42	2.9E7
INDFVNGCRQVW	77.40	1.42E8
GKSHMLSPEIVQDISEIEENCEPF	76.85	6.86E7
AINMHAVEFDESPEKLW	76.81	4.08E7
SHPQFEKGSSMASMSDSEVNQEAKPEVKPEVKPETHINL	76.50	1.05E8
SHPQFEKGSSMASM	76.41	4.9E6
DGIRIQADQTPEDLDMEDNDIIEAHREQ	76.07	1.76E7
IVPRKKEIAEKA	75.99	1.33E7
AMQLTDKSRNELF	75.70	5.34E7
AVEFDESPEKLW	75.68	3.32E8
AHREQIGGIEENLY	75.44	1.29E7
KMMEEKAINMH	75.42	1.03E6
YFLPGGVKKSGAVRVSL	75.35	3.78E7
SRIKQEASYH	75.32	2.54E8
LYDGIRIQADQ	75.29	1.27E7
IDIHKISENENILK	75.17	5.07E6
FLPGGVKKSGAVR	75.11	3.63E8
RFPWYELYSDYSEN	74.65	2.62E7
ALDTIFEKTIGW	74.64	2.87E7
KISENENILKTL	74.24	2.1E8
LPGGVKKSGAVR	74.18	5.72E7
LNSVEKIRRQVEF	73.96	3.08E8
KIAFEADEICW	73.85	2.68E6
DKVEERIKKIDIHKISENENILKTL	73.83	9.41E7
LLDKVEERIKKIDIH	73.59	8.08E6
KLTREIPQEY	73.54	8.31E7

EKLTREIPQEYLHEISLEL	73.15	1.37E7
LLSRIKQEASY	73.14	4.35E9
TREIPQEYLHEISLEL	73.07	2.79E7
MGASNSVEKY	73.07	1.5E8
FLIVPRKKEIAEKAM	72.99	2.08E7
RLVVLNESRRY	72.89	5.39E8
DYFLPGGVKKSGAVR	72.80	5.83E6
KKEIAEKAMKAL	72.74	4.45E7
LPGGVKKSGAVRV	72.63	8.42E6
LIVPRKKEIAEK	72.54	4.9E8
SLLDKVEERIK	72.47	2.02E9
KIDIHKISEN	72.46	6.02E6
DYFLPGGVKK	72.35	2.05E7
MKALDTIFEKTIGW	72.34	3.74E6
YHLEQIPEDERY	72.11	5.25E6
KFLAAFC	71.97	7.28E7
KGRMGASNSVEKY	71.94	4.36E7
RFPWYELYSDYSENTL	71.57	1.01E7
CGPNLDDKQRLS	71.51	7.84E5
ASMSDSEVNQEAK	71.31	5.25E5
SEVNQEAKPEVKPEVKPETHINL	71.07	1.47E8
MHAVEFDESPEKL	70.90	1.3E6
KSWFEGNC	70.66	5.02E6
KALDTIFEKT	70.51	1.09E6
LAAFCGPNLDDKQRL	70.27	7.2E7
LLNSVEKIRR	70.06	4.61E8
NSVEKIRRQVEF	70.05	3.98E8
KALDTIFEKTIG	70.02	9.9E6
LYDGIRIQADQTPED	69.98	8.1E6
KRVVLGEGVEDYEKIW	69.77	6.36E6
INLKVSDGSSEIFF	69.77	9.29E8
TREIPQEYLHEISL	69.65	4.84E7
KAINMHAVEF	69.27	1.08E7
GRMGASNSVEKY	69.00	2.3E7

IKKIDIHKISENENILKTL	68.81	9.88E7
RIKKIDIHKISENENIL	68.66	9.9E6
RLSLLDKVEERIK	68.37	2.1E5
EIAEKAMKALDTIF	68.36	3.72E6
RMGASNSVEKY	68.33	1.74E7
MEEKAINMHAVEFDESPEKL	68.30	1.78E6
KPEVKPEVKPETHINL	68.27	5.88E7
KIDIHKISENE	67.80	1.51E7
QKGRMGASNSVEKY	67.73	2.18E8
IKKIDIHKISENENIL	67.64	1.24E8
ELQKGRMGASN	67.57	2.17E7
KLIEDAKIAFE	67.35	8.19E6
IDIHKISENENILKTL	67.22	4.52E7
KLIEDAKIAFEA	67.21	8.39E6
IVPRKKEIAEK	67.17	6.06E7
IKMMEEKAINMHAVEFDESPEKL	67.14	3.39E7
TNIELVTATDED	66.96	6.53E6
LLDKVEERIK	66.79	3.95E7
KVSDGSSEIFF	66.67	4.59E9
KPEVKPETHINL	66.62	6.39E6
SHPQFEKGSSMA	66.56	5.07E7
RLLAAVSKPSSL	66.43	7.41E5
LSPEIVQDISEIEENCEPF	66.21	1.05E8
RFPWYELYSDY	66.20	8.21E7
FEGNCDNAKL	66.10	1.76E8
SVEKIRRQVEF	65.92	1.69E9
HDAEVSFIAF	65.86	3.08E8
DEDSLWHDAEVSF	65.70	1.77E6
KLIEDAKIAF	65.58	7.52E8
GASNSVEKY	65.21	5.49E7
KALDTIFEK	65.14	5.55E7
FEGNCDNAKLI	65.04	3.35E6
SLWHDAEVSF	64.99	2.55E7
MLSPEIVQDISEIEENCEPF	64.83	6.03E8

IEDAKIAFEADEICW	64.78	5.48E6
EVKPEVKPETHINL	64.76	9.63E6
EKGGGSGGGSGGGAW	64.70	7.38E7
GVKKSGAVRVSL	64.69	1.87E8
QLTDKSRNELF	64.45	9.9E8
MEEKAINMH	64.42	5.58E6
PEVKPETHINL	63.95	1.67E7
SHPQFEKGGGSGGG	63.70	6.79E5
INLKVSDGSSEIF	63.41	2.36E8
QFLIVPRKKEIAEK	63.39	2.49E6
DNDIIEAHREQIGGIEENLY	63.37	8.57E6
DYFLPGGVKKS	63.15	5.77E6
DIHKISENENILKTL	63.09	1.99E6
KISENENILK	62.98	8.32E7
DGIRIQADQTPEDL	62.88	4.28E6
HREQIGGIEENLY	62.86	2.11E7
DGIRIQADQTPEDLDMEDNDIIEAH	62.83	9.8E6
VKPEVKPETH	62.79	2.1E6
SWFEGNCDNAKL	62.74	2.89E7
TNIELVTATDEDSLWHDAEVSF	62.66	6.18E8
IKMMEEKAIN	62.43	1.03E8
DIHKISENENIL	62.39	4.92E6
FDESPEKLW	62.35	8.76E7
EGNCDNAKL	62.23	1.01E8
IAEKAMKALDTIF	62.10	1.11E7
LSRIKQEASY	62.07	2.44E7
INDFVNGCRQV	62.06	2.08E8
LIVPRKKEIAEKAMKALDTIF	62.06	2.51E8
ASMSDSEVNQEAKPEVKPEVKPE	61.96	1.32E7
LPGGVKKSGAV	61.96	3.77E6
TLKSWFEGN	61.90	3.54E6
DIHKISENENILK	61.75	2.32E6
PQFEKGSSM	61.71	4.52E5
HAVEFDESPE	61.20	5.55E6

KAINMHAVEFDESPEKL	61.16	1.45E6
KLIEDAKIAFEAD	61.11	1.59E7
AKRQGKEMDSL	60.92	1.42E9
FLPGGVKKSGAVRV	60.87	1.72E8
HLEQIPEDERYR	60.82	1.98E7
PNLDDKQRL	60.71	1.28E7
REQIGGIEENLY	60.45	2.94E7
QFLIVPRKKEIAEKAM	60.31	2.34E7
FAKRQGKEMDSL	60.04	1.58E7
IDIHKISENENIL	59.86	3.5E7
VEERIKKIDIH	59.81	1.22E5
SLLDKVEERIKKI	59.79	4.11E7
KKEIAEKAM	59.75	6.08E7
MQLTDKSRNELF	59.71	4.98E6
PGGVKKSGAVRV	59.67	1.34E7
AMKALDTIFEKTIGW	59.49	1.77E7
VKPEVKPETHINL	59.39	3.03E8
SMASMSDSEVNQEAKPEVKPEVKPETHINL	59.39	3.13E7
LTREIPQEY	59.35	1.46E7
VNQEAKPEVKPEVKPETHINL	59.35	4.24E6
TNIELVTATDEDSLWH	59.28	2.75E6
AAVSKPSSL	59.25	7.86E9
SSMASMSDSEVNQEAKPEVKPEVKPETH	59.03	4.3E6
LIVPRKKEIAEKAMKALD	58.97	3.32E6
LYDGIRIQAD	58.90	1.2E7
TNIELVTATDEDSLW	58.86	5.7E8
HLEQIPEDE	58.85	1.24E7
DRVNYLMEVL	58.72	6.31E6
NAIMELQKGRMGA	58.71	8.14E6
HLEQIPEDER	58.71	1.05E7
KSTVDEPSVLY	58.70	2.57E9
GPNLDDKQRL	58.70	7.87E7
IIEAHREQIGGIEENLY	58.24	1.36E7
LLSRIKQEAS	57.87	1.73E6

EWGKSHM	57.84	1.7E7
AVEFDESPEKL	57.66	1.5E7
MEEKAINM	57.52	1.3E7
RLGDEAALDYFLPGGVKK	57.43	6.04E6
VVLNESRRY	57.41	1.64E8
IAFDRVNYL	57.39	8.14E6
HPQFEKGSSM	57.38	1.07E7
REQIGGIEENLYF	57.37	1.65E6
IHKISENENILKTL	57.34	1.47E7
AWSHPQFEKGSSM	57.30	8.43E5
LYDGIRIQADQTPEDLD	57.04	3.42E6
LIVPRKKEIAE	57.03	2.77E7
NAIMELQKGRMGASN	56.92	2.17E6
KSWFEGN	56.90	4.63E7
SMSDSEVNQEAKPEVKPEVKPETH	56.88	2.34E7
IAEKAMKAL	56.81	2.97E6
NAIMELQKGRMGASNSVEKY	56.79	5.42E7
SGAVRVSL	56.78	1.35E9
ISENENILKTL	56.71	8.68E5
GGVKKSGAVRVSL	56.60	2.32E8
EKTIGWLEESEFSPL	56.51	9.9E8
YFLPGGVKK	56.48	4.74E6
KIDIHKISE	56.22	1.5E6
KVEERIKKIDIH	56.21	9.74E6
QIFKSTVDEPSVLY	56.15	3.18E8
NAIMELQKGRM	56.10	5.58E6
KVSDGSSEIF	56.10	1.14E9
DESPEKLW	55.97	4.9E8
TDKSRNELF	55.94	1.96E9
EADEICWKFL	55.91	1.06E6
VKKSGAVRVSL	55.76	6.68E6
LTDKSRNELF	55.75	4.42E7
NMHAVEFDESPEKLW	55.73	6.98E6
INDFVNGCR	55.71	7.5E7

IKMMEEKAINMHA	55.63	1.19E6
FKIKKTTPL	55.62	2.94E8
RQGKEMDSLRF	55.58	4.5E6
SLLDKVEER	55.57	1.83E8
KGSSMASMSDSEVNQEAKPEVKPEVKPETHINL	55.55	3.5E6
LEQIPEDERY	55.40	2.45E7
NSVEKIRR	55.40	1.34E7
IAFDRVNY	55.31	1.88E9
DESPEKL	55.28	5.27E7
DSLWHDAEVSF	55.08	1.83E7
AKIAFEADEICW	54.64	1.01E7
PFIKSDEF	54.62	2.04E6
CGPNLDDKQR	54.52	1.59E6
LKLIEDAKIAF	54.41	8.01E6
PGGVKKS	54.37	2.28E6
TIFEKTIGW	54.32	5.28E7
RLGDEAALDYFL	54.22	4.17E7
YGARAIAA	54.19	1.71E8
QFLIVPR	54.13	2.47E7
EVNQEAKPEVKPEVKPETHINL	54.08	2.00E7
ERIKKIDIHKISENENIL	54.06	6.75E6
IKKIDIHKISENENILK	54.05	7.35E6
RLGDEAALDYF	53.96	1.23E8
AINMHAVEF	53.85	6.39E6
AFDRVNY	53.70	2.97E7
FVNGCRQVW	53.69	1.69E5
RLGDEAALDY	53.69	3.96E9
KTIGWLEESEFSPL	53.65	1.72E7
FQSNAMQLTDK	53.60	2.17E6
SLWHDAEVSFIAF	53.55	8.41E5
LLNSVEKIR	53.41	1.51E8
LAAVSKPSSL	53.40	1.04E9
LLAAVSKPSSL	53.36	1.76E7
SDYSENTLEIIIENF	53.29	2.28E7

EWGKSHML	53.28	5.42E7
MLSPEIVQDISEIEEN	53.27	3.39E6
KSWFEGNCD	53.19	3.59E6
GKEMDSLRF	53.11	6.26E6
QSNAMQLTDKSRNELF	53.04	9.25E5
TNIELVTATDE	52.74	2.38E6
DSEVNQEAKPEVKPEVKPETHINL	52.73	8.59E6
IKQEASYH	52.72	1.2E7
NAIMELQKGR	52.68	9.13E6
TNIELVTATDEDSL	52.60	2.41E6
AKPEVKPEVKPETHINL	52.50	3.00E7
EKLTREIPQEYLHEISL	52.48	7.03E6
AHREQIGGIEENLYF	52.38	9.53E5
LDKVEERIK	52.36	1.1E7
IKSDEFTNF	52.34	4.94E9
EGNCDNAKLI	52.11	2.35E6
IVQDISEIEENCEPF	52.08	6.68E7
DTIFEKTIGW	51.98	1.45E8
LYDGIRIQADQTPEDL	51.91	5.32E6
LSGKWEKL	51.88	3.82E8
FEGNCDNAKLIKISF	51.79	1.27E5
TREIPQEY	51.76	7.36E9
FLPGGVKKSGAV	51.76	7.02E7
SLLDKVEERIKK	51.72	3.7E7
DRVNYLMEVLQF	51.68	1.09E6
HDAEVSFI	51.60	2.23E7
LYDGIRIQ	51.46	7.54E6
KEMDSLRF	51.11	5.84E6
KAMKALDTIF	51.02	6.48E6
KLIEDAKIAFEADEIC	50.93	3.02E6
EKTIGWLEESEF	50.87	9.03E8
EAKPEVKPEVKPETHINL	50.82	1.14E7
SLLDKVEERIKKID	50.78	1.48E7
VNGCRQVW	50.76	2.34E7

PRKKEIAEK	50.72	2.43E6
SRIKQEASY	50.66	1.35E9
TIGWLEESEFSPL	50.63	1.3E7
QIPEDERY	50.53	2.76E7
IVPRKKEIAEKAMKALDTIF	50.42	6.65E7
HDAEVSFIAFD	50.26	9.44E5
EQIPEDERY	50.17	4.23E7
EIAEKAM	50.14	1.96E8
LLSRIKQEA	50.12	1.17E7
ERIKKIDIHKISENENILKTL	50.05	4.36E6
EWGKSHMLSPEIVQDISEIEENCEPF	50.02	1.49E8
MMEEKAINM	50.00	6.33E5
KISENENIL	49.98	6.23E8
EEKAINMHAVEFDESPEKLW	49.84	1.03E7
EKLTREIPQEYL	49.83	1.82E6
SLLDKVEERI	49.64	3.69E6
HLEQIPED	49.55	2.36E7
ASMSDSEVNQEAKPEVKPEVKPETHIN	49.49	6.61E6
SHPQFEK	49.46	4.56E6
CGPNLDDKQ	49.33	7.89E6
ELQKGRMGA	49.32	5.4E7
NAIMELQK	49.11	1.24E7
KLIEDAKIAFEADEI	49.07	2.46E6
PFIKSDEFTNF	49.05	2.22E7
SGKWEKL	49.00	2.16E8
KALDTIF	48.83	9.77E8
YRFPWYELY	48.81	1.02E6
KIDIHKIS	48.73	1.34E6
TNFLLNSVEK	48.66	7.88E5
EDSLWHDAEVSF	48.63	2.18E7
SDEFTNF	48.55	1.54E7
SHPQFEKGSSMASMSDSEVNQEAKPEVKPEVKPE	48.37	2.74E6
RFPWYELY	48.30	4.5E9
EIIIENFDTF	48.26	1.52E8

NAIMELQKGRMG	48.24	5.29E5
ERIKKIDIH	48.11	5.63E6
QIFKSTVDEPSVL	48.10	2.83E7
TDKSRNEL	48.06	3.83E7
NQEAKPEVKPEVKPETHINL	48.01	8.12E5
SMASMSDSEVNQEAKPEVKPEVKPETH	47.96	3.78E6
DFVNGCRQVW	47.78	3.02E6
IPQEYLHEISLEL	47.67	4.28E5
AMQLTDKSRNEL	47.66	4.95E5
NAIMELQKG	47.62	8.82E6
QSNAMQL	47.57	1.13E7
AVSKPSSL	47.56	1.17E7
DYFLPGGVK	47.47	1.77E7
LIEDAKIAF	47.47	5.35E6
NSVEKIR	47.42	9.51E6
DGIRIQAD	47.38	3.4E6
SDYSENTLEIIIENFDTF	47.29	1.49E8
FLPGGVKKSGA	47.12	1.5E8
TLEIIIENFDTF	46.86	3.7E7
DKVEERIK	46.83	3.52E7
ISENENILK	46.79	1.23E6
FEKTIGW	46.66	7.14E5
DGIRIQADQ	46.56	7.5E6
LLNSVEK	46.44	2.7E9
LLDKVEER	46.39	2.24E6
LDKVEER	46.38	6.08E6
PETHINL	46.37	1.22E7
TPEDLDMEDNDIIEAHREQIGGIEENLY	46.30	9.89E6
RQGKEMDSL	46.30	8.24E6
IFEKTIGW	46.29	1.69E7
EVKPETHINL	46.27	4.73E6
EADEICW	46.15	1.06E7
FLPGGVKKSGAVRVS	45.99	1.72E7
EIAEKAMK	45.91	8.25E6

RRLMEAF	45.73	3.58E9
RIKQEASY	45.72	1.23E7
DTFLSGKW	45.45	6.68E6
QEAKPEVKPEVKPETHINL	45.25	7.71E6
DKVEERIKKI	45.12	1.24E7
KLELNNNPILL	45.04	3.01E5
SHPQFEKGSSMASMSD	45.03	2.61E5
KSWFEGNCDNAKLIKISF	45.02	3.11E6
SNSVEKY	44.96	8.04E6
QLTDKSRNEL	44.91	5.1E6
AMKALDTIF	44.90	2.59E8
TIGWLEESEF	44.68	2.73E6
TREIPQEYL	44.64	4.2E6
KISFDTW	44.60	1.73E7
TDKSRNELFSALL	44.55	2.49E7
HDAEVSF	44.54	1.55E9
SDYSENTL	44.54	7.27E7
FLPGGVKKS	44.42	1.04E8
AFCGPNLDDKQRL	44.08	7.48E5
KLWRLGDEAALDY	44.02	1.79E6
TLEIIIENF	44.01	2.69E6
SPLRLVVL	43.86	9.48E6
HAVEFDESPEKLWNAIM	43.76	1.24E6
EIAEKAMKAL	43.69	6.73E6
KLIEDAK	43.56	1.27E7
SLLDKVE	43.56	2.02E7
IKISFDTW	43.52	1.64E9
KIKKTTPLR	43.47	1.19E6
INLKVSD	43.44	3.96E6
LHEISLEL	43.32	3.83E9
IPEDERY	43.08	2.98E6
FQSNAMQL	43.06	2.03E8
SLKPNPKGEY	43.00	7.42E6
FLPGGVKK	42.91	8.09E8

QGKEMDSLRF	42.84	3.43E5
IGGIEENLYF	42.77	1.37E6
LLNSVEKI	42.71	3.05E7
NENILKTL	42.67	1.36E6
INDFVNGC	42.44	2.52E6
LEESEFSPL	42.34	3.88E8
EMDSLRF	42.17	3.12E6
FQSNAMQLT	42.14	2.66E6
LYDGIRIQADQTPE	42.10	5.16E6
DYFLPGGVKKSGAVRV	42.09	2.73E6
LEWGKSH	42.08	1.94E7
LIVPRKKE	41.99	1.18E7
AAFCGPNLDDKQ	41.98	2.2E6
GNCDNAKL	41.86	1.24E6
IAFEADEICW	41.76	4.52E6
LSLLDKVEERIK	41.73	3.8E5
DTIFEKTIGWL	41.65	3.14E5
KLIEDAKI	41.53	6.04E6
EAFAKRQGKEMDSL	41.28	5.53E6
VNQEAKPEVKPEVKPETH	41.23	2.15E6
EFIQELPAVIF	40.93	3.01E5
SDSEVNQEAKPEVKPEVKPE	40.76	1.3E6
LLNSVEKIRRQ	40.76	1.81E6
AFEADEICW	40.71	1.42E6
LYDGIRIQADQTPEDLDMEDNDIIEAHREQIGGIEENL	40.65	2.69E6
FPWYELY	40.65	8.27E6
LYDGIRIQADQTPEDLDMEDNDIIEAHREQIG	40.62	7.21E6
ELQKGRMG	40.59	3.69E6
KLWRLGDEAAL	40.59	1.39E6
FIKSDEFTNF	40.56	1.62E7
SVEKYINDF	40.49	3.46E6
MEEKAIN	40.41	5.75E6
KALDTIFEKTIGWL	40.31	5.23E5
GSSMASMSDSEVNQEAKPEVKPEVKPETHINL	40.30	7.29E6

TDEDSLW	40.28	1.21E6
KIKKTTPL	40.28	8.02E8
VLNESRRY	40.27	2.35E6
LDDKQRL	40.17	3.01E7
WRLGDEAALDY	40.08	4.31E5
FIAFDRVNY	40.08	3.8E6
TDEDSLWHDAEVSF	39.88	1.21E7
HLEQIPE	39.85	9.47E6
FCGPNLDDKQRL	39.65	3.3E6
LKVSDGSSEIFF	39.61	3.3E7
SNAMQLTDKSRNELF	39.60	1.21E6
KSTVDEPSVLYG	39.56	3.94E7
ELQKGRM	39.54	3.25E6
DGIRIQADQTPEDLD	39.51	8.6E5
DKQRLSLLDKVEERIK	39.45	1.04E6
ITNDDKPDVASVQQIENRIL	39.36	7.48E5
IKMMEEKA	39.31	2.00E7
IEDAKIAF	39.23	1.02E7
SHPQFEKGSSMASMSDSEVN	39.02	3.95E6
HAVEFDE	38.99	3.93E6
VSKPSSL	38.93	6.17E7
KSSSAW	38.89	4.49E8
DEAALDY	38.84	7.4E6
THINLKVSDGSSEIFF	38.80	4.74E6
FLLNSVEK	38.58	9.2E5
SHPQFEKGSSMASMSDSEVNQEAKPE	38.54	1.42E6
ELKRDKLL	38.46	4.88E7
GKEMDSL	38.45	9.35E6
ESEFSPL	38.41	2.82E6
ASMSDSEVNQEAKPEVKPEVK	38.35	5.05E5
LAAVSKPS	38.27	6.73E7
EIVQDISEIEENCEPF	38.23	2.64E5
IRRQVEF	38.04	1.75E7
KTLKSWF	38.02	1.88E7

TREIPQEYLH	38.00	6.44E5
INDFVNG	38.00	6.48E7
EKTIGWLEES	37.96	7.46E6
EKTIGWLEESE	37.95	4.52E6
RLVVLNESRR	37.83	1.84E6
RLVVLNE	37.82	3.88E6
IKKIDIH	37.60	1.59E7
HAVEFDESPEKLWN	37.59	1.4E6
KVEERIKKIDIHKISENENIL	37.45	2.05E6
KSGAVRVSL	37.31	9.56E8
YFQSNAM	37.30	2.08E6
RLGDEAAL	37.30	3.87E8
LMEVLQFL	37.07	2.66E7
IKSDEFTNFLLN	37.03	1.99E6
NAIMELQ	37.02	3.99E7
SENTLEIIIENFDTF	36.90	1.11E7
EYLHEISLEL	36.84	4.79E6
DESPEKLWNAIM	36.78	1.68E6
LLSRIKQ	36.78	1.89E7
DGIRIQADQTPEDLDMEDNDIIEAHREQIG	36.72	1.95E6
SVEKIRR	36.71	5.6E7
YDGIRIQADQTPEDLDMEDNDIIEAHREQIGGIEENLY	36.65	3.9E6
WFEGNCDNAKL	36.62	2.18E6
IKSDEFTNFL	36.56	8.21E6
RFPWYEL	36.55	2.78E7
DGIRIQA	36.50	2.54E6
ASMSDSEVNQEAKPE	36.37	8.63E5
IKMMEEKAINMHAVEFDE	36.33	4.49E6
ELNNNPILL	35.94	2.7E6
RLGDEAALDYFLPGGVK	35.92	7.96E5
MASMSDSEVNQEAKPEVKPEVKPETHINL	35.90	5.36E6
LIVPRKKEIA	35.81	1.99E6
DGIRIQADQTPEDLDMEDNDIIEAHREQIGGIEENLYF	35.64	1.03E7
ELKRDKL	35.64	2.49E8

GIEENLYF	35.57	1.04E6
LDKVEERIKKIDIHKISENENIL	35.56	4.38E6
DKSRNELF	35.53	1.58E7
EFDESPEKLW	35.42	1.18E6
ISEIEENCEPF	35.36	2.02E6
KVEERIK	35.28	1.06E6
LIKISFDTW	35.20	2.59E6
NLKVSDGSSEIFF	35.19	1.92E6
TIFEKTIGWLEESEF	35.16	1.49E7
IGWLEESEFSPL	35.09	1.23E6
EIIIENFDTFL	35.03	7.25E5
EKTIGWLEE	34.97	1.01E7
AAFCGPNLDDKQRLSLL	34.87	1.21E6
LNSVEKIRR	34.79	9.13E5
IRIQADQTPEDLDMEDNDIIEAHREQIGGIEENLY	34.73	1.66E6
IIIENFDTF	34.70	4.55E7
DTIFEKTIGWLEE	34.59	1.28E6
AAFCGPNLDDKQR	34.56	8.77E5
KIKKTTP	34.46	9.47E5
IEDAKIAFEADEICWKF	34.33	4.01E6
HAVEFDESPEKLWNAIMEL	34.22	2.43E6
RLVVLNES	34.10	1.2E7
SLLDKVEE	34.04	1.85E7
PEVKPETHINLKV	33.93	2.53E8
LPGGVKKS	33.91	3.43E6
VKPETHINL	33.90	9.19E7
LWHDAEVSF	33.69	1.47E6
KIRRQVEF	33.55	1.93E7
MLSPEIVQDISEIEENC	33.51	6.51E5
EEKAINM	33.09	4.25E7
RFPWYELYS	33.08	4.52E6
EYLHEISL	32.98	1.38E6
GGGSGGGSGGGAW	32.94	1.68E7
INDFVNGCRQ	32.85	2.14E6

HKISENENILKTL	32.83	9.16E5
SENENILKTL	32.78	2.65E6
ELKRDKLLL	32.64	1.72E8
HREQIGGIEENLYF	32.50	6.63E5
DAEVSFIAF	32.40	3.21E5
HEISLEL	32.34	2.25E7
LYDGIRIQADQTPEDLDM	32.27	1.05E6
DTIFEKTIGWLEESEFSPL	32.12	1.15E7
HMLSPEIVQDISEIEENCEPF	31.63	1.69E7
DSLRFLY	31.49	1.27E6
YLMEVLQF	31.29	5.86E5
ALKRVVLGEGVEDYEKIW	30.98	1.24E6
LHEISLE	30.94	9.29E6
KSRNELF	30.72	5.24E6
GSSEIFF	30.64	4.21E7
KRLLAAV	30.61	2.62E6
TFLSGKW	30.61	4.04E6
DKSRNEL	30.35	2.66E6
LMEVLQF	30.26	6.4E8
TIFEKTIGWLEESEFSPL	30.24	5.93E6
DMEDNDIIEAHREQIGGIEENLY	30.15	4.43E6
EGKGPLKDSLVK	30.05	1.12E5
YDGIRIQ	29.97	2.54E5
IELVTATDEDSLW	29.91	1.65E6
DGIRIQADQTPEDLDM	29.90	3.09E5
LVVLNESRRY	29.83	9.97E5
ESPEKLW	29.83	4.09E6
EKGSSMASMSDSEVNQEAKPEVKPEVKPETHINL	29.81	2.49E6
IKSDEFTNFLL	29.54	3.53E6
NIELVTATDEDSLW	29.45	4.21E5
LVTATDEDSLW	29.29	5.85E6
EPFIKSDEFTNF	29.20	7.18E6
DGSSEIFF	29.16	7.63E5
LGDEAAL	29.14	1.72E6

TDKSRNELFSALLEWGKSH	29.09	4.18E6
SLLDKVEERIKKIDIHK	28.98	6.66E5
PETHINLKV	28.95	9.19E7
MKALDTIF	28.87	2.14E7
WHDAEVSF	28.82	7.84E6
YLHEISL	28.76	5.56E5
TREIPQE	28.61	1.51E7
STVDEPSVLY	28.56	1.26E6
SSEKSVQPR	28.53	4.78E5
LYDGIRIQADQTPEDLDME	28.47	2.37E5
VTATDEDSLWHDAEVSF	28.41	6.84E6
FQSNAMQLTDKSRNEL	28.23	3.15E6
GEGVEDYEKIW	28.16	8.83E5
IQELPAVIF	28.15	1.16E6
FLPGGVKKSG	27.94	2.38E7
IAFDRVNYLMEVLQF	27.86	4.00E5
KSDEFTNFLL	27.77	3.00E7
SRIKQEASYHKR	27.74	1.02E7
GIEENLY	27.65	1.62E7
SLKPNPKGEYIIL	27.65	1.61E6
IFKSTVDEPSVLY	27.51	8.3E6
KLIKISF	27.51	3.23E6
EIPQEYLHEISLEL	27.38	2.59E6
PLRLVVL	27.35	4.01E6
RLGDEAALD	26.97	2.26E6
LKVSDGSSEIF	26.90	6.43E6
PRKKEIAEKA	26.70	9.76E4
MLSPEIVQDI	26.66	3.83E6
KLIEDAKIAFEADE	26.49	7.3E5
KGRMGASNSVEKYINDFVNGCRQVW	26.41	5.61E6
KRDKLLL	26.38	1.44E9
IQADQTPEDLDMEDNDIIEAHREQIGGIEENLY	26.28	3.67E6
LKRDKLLL	26.07	7.19E6
MSDSEVNQEAKPEVKPEVKPETH	25.91	5.07E5

FIKSDEF	25.84	1.4E6
HKRLLAAVSKPSSL	25.65	6.02E5
LRRLMEAF	25.46	1.61E5
AIMELQKGRMGASNSVEKY	25.41	9.44E5
RLVVLNESRRYH	25.40	6.59E5
ENILKTL	25.35	3.94E6
SHPQFEKGGGSGGGSGGGA	25.12	1.85E6
PEDLDMEDNDIIEAHREQIGGIEENLY	25.02	5.56E5
GARAIAA	24.99	7.86E6
GWLEESEFSPL	24.96	1.05E6
KLIKISFDTW	24.85	2.02E5
FEGNCDNAKLIKISFDTW	24.58	1.44E6
IKMMEEKAI	24.42	3.66E6
NIELVTATDEDSLWHDAEVSF	24.38	2.16E5
EDNDIIEAHREQIGGIEENLY	24.34	1.14E7
LDKVEERIKKIDIHKISENENI	24.32	3.45E6
LIVPRKK	23.95	4.28E7
FTNIELVTATDEDSLW	23.93	8.07E5
YELYSDYSEN	23.64	1.52E6
YLHEISLEL	23.45	1.92E6

Table S4. Mass spectrometry analysis of the chymotrypsin-treated small Csx30 fragment. In order to identify the *Jc*-Craspase cleavage site, no assumption of cleavage site preference was made in peptide sequence mapping.

Peptides	-10 log P	Area
ITNDDKPDVASVQQIENRILA	109.76	1.53E9
ITNDDKPDVASVQQIENRI	93.75	1.59E7
CGCFITNDDKPDVASVQQ	91.29	1.7E8
ITNDDKPDVASVQQIENRILAK	89.23	6.49E7
CGCFITNDDKPDVAS	89.03	4.36E6
KRVVLGEGVEDYEKIWN	88.64	1.14E9
SSEKSVQPRKLE	86.61	3.61E9
ALKRVVLGEGVEDYE	85.81	4.2E8

KPDVASVQQIENRILA	84.98	1.83E7
RVVLGEGVEDYEKIWN	83.70	3.46E7
ALKRVVLGEGVEDYEKIWN	80.41	9.9E7
SSEKSVQPRK	80.09	5.71E8
ITNDDKPDVASVQQIENR	80.05	8.07E8
KRVVLGEGVEDYEKIW	80.01	2.8E6
KKAIIGVSPEKIVLE	79.36	1.41E8
PDVASVQQIENRILA	79.30	2.04E6
FITNDDKPDVASVQQ	78.90	1.33E8
KKAIIGVSPEKIVLEE	78.59	4.34E7
ITNDDKPDVASVQQ	77.86	2.29E9
SEKSVQPRKLE	77.50	5.78E7
FITNDDKPDVASVQQIENRILA	76.48	1.78E7
NYLEHTKNDYWC	75.69	4.88E8
DDKPDVASVQQIENRILA	75.64	2.59E7
EGKGPLKDSLVKKV	75.04	1.36E8
SLKPNPKGEYI	74.26	4.7E9
KRVVLGEGVEDYE	73.55	6.33E8
KPDVASVQQIENR	73.48	1.37E7
NYLEHTKNDYW	72.70	1.38E8
CGCFITNDDKP	72.62	7.28E5
DKPDVASVQQIENRILA	72.47	1.55E7
EGKGPLKDSLVKK	72.36	1.4E9
SSEKSVQPRKLEL	71.92	2.47E6
DVASVQQIENRILA	71.17	1.94E6
NYLEHTKNDYWCGCFI	69.06	3.44E7
YLEHTKNDYWC	68.30	2.19E7
GEGVEDYEKIWN	68.20	5.33E8
LSLKPNPKGEYI	67.83	1.02E8
ITNDDKPDVASVQQI	67.58	1.59E7
VASVQQIENRILA	67.48	8.31E6
CGCFITNDDKPDVASVQQIENRILA	66.99	1.58E7
SVQQIENRILA	66.72	1.43E8
SLKPNPKGEYIILS	65.56	1.67E9

ITNDDKPDVASVQ	65.45	4.81E6
VVLGEGVEDYEKI	65.33	2.37E5
RILAKKTRDYK	64.96	1.56E7
SEKSVQPRK	64.38	3.17E7
GPLKDSLVKK	64.20	2.73E6
KKAIIGVSPEKIV	63.94	4.27E6
EGKGPLKDSLVK	63.85	2.05E7
DDKPDVASVQQIENR	63.63	1.18E7
NYLEHTKNDY	63.19	2.38E7
ASSEKSVQPRKLE	63.18	7.92E4
ASVQQIENRILA	63.07	3.67E6
AASSEKSVQPRK	62.96	1.06E6
NYLEHTKNDYWCGCF	61.89	4.79E6
LKRVVLGEGVEDYE	61.50	8.94E5
LEHTKNDYWC	61.05	9.64E7
KRVVLGEGVEDY	60.79	1.74E7
ITNDDKPDVAS	60.35	1.83E8
IIGVSPEKIVLE	59.91	6.81E6
LEHTKNDYW	59.68	4.68E6
DKPDVASVQQIENR	59.59	9.75E6
CGCFITNDDKPDV	59.52	1.32E6
KAIIGVSPEKIVLE	59.21	3.89E6
AIAASSEKSVQPRKLE	59.14	3.49E6
SLKPNPKG	59.05	1.24E7
ALKRVVLGEGV	58.41	6.67E6
KLELNNNPILLS	58.39	7.4E8
KDSLVKKVIILV	58.27	1.88E8
KPNPKGEYIILS	58.22	4.04E7
KSTVDEPSVLYG	57.80	2.65E7
EGKGPLKDSLV	57.48	4.12E8
NYLEHTKND	57.02	1.3E8
KRVVLGEGVEDYEKI	56.89	5.62E6
YLEHTKND	56.89	3.48E6
EKSVQPRKLE	56.75	4.53E6

VLGEGVEDYEKIWN	56.40	2.46E6
KKAIIGVSPEKIVLEEFIQELPAVIFE	56.32	3.2E7
EHTKNDYW	56.24	3.18E7
SLKPNPKGEYIILSSLA	56.04	1.96E7
VVLGEGVEDYEKIWN	55.93	9.5E7
EHTKNDYWCGCFI	55.92	2.00E7
EHTKNDYWC	55.82	2.82E8
KKAIIGVSPEKIVLEEFI	55.47	1.6E7
GVEDYEKIWN	55.38	2.93E7
PDVASVQQIENR	55.08	8.43E5
RVVLGEGVEDYE	55.02	1.28E7
EEFIQELPAVIFE	54.83	2.97E6
EFIQELPAVIFE	54.54	1.48E8
GCFITNDDKPDVAS	54.27	7.79E5
SVQPRKLE	54.10	2.18E8
DESPEKLWN	53.93	2.7E6
GKGPLKDSLV	53.89	3.75E6
EGVEDYEKIWN	53.50	5.16E7
SVQQIENRI	53.49	2.51E6
KKAIIGVSPEK	53.46	1.78E8
KPNPKGEYI	53.10	8.16E7
SLKPNPKGE	52.88	4.74E6
SVQQIENRILAK	52.52	1.57E6
PNPKGEYI	52.17	2.43E7
KGPLKDSLVKK	52.06	8.11E5
ELNNNPILLS	51.87	2.45E9
RKLELNNNPILLS	51.78	1.38E7
SLKPNPKGEY	51.63	2.23E6
KSWFEGNCDNAKLI	51.55	6.95E6
KIVLEEFI	51.42	9.8E7
GKGPLKDSLVKK	51.38	9.29E5
VIFEGKGPLK	51.36	2.34E6
ALKRVVLGEGVED	50.96	2.25E6
DDKPDVASVQQ	50.81	5.34E7

GEGVEDYEKIW	50.73	1.64E6
CGPNLDDKQRLS	50.68	3.07E6
LELNNNPILLS	50.46	2.2E7
AKRQGKEMDSLR	50.22	5.67E6
DKPDVASVQQ	50.17	2.27E7
KAIIGVSPEK	49.84	4.04E6
NDDKPDVASVQQIENRILA	49.45	1.65E6
AKKTRDYK	49.35	4.48E8
GEGVEDYEKI	49.27	3.00E5
ALKRVVLGEGVE	49.21	5.44E5
ILLSLKPNPKGEYI	48.85	3.98E6
IQELPAVIFE	48.48	9.2E8
KDSLVKKV	48.13	2.6E6
KSVQPRKLE	47.71	8.63E6
HTKNDYWC	47.35	1.75E6
CGCFITNDD	47.32	6.4E5
EKSVQPRK	46.61	6.59E7
GVSPEKIVLEEFI	46.56	7.51E6
KVSDGSSEIFF	46.36	6.02E6
ITNDDKPDV	46.28	8.82E6
LAKKTRDYK	46.21	2.93E6
AIAASSEKS	46.13	1.23E6
LKPNPKGEYI	46.13	4.08E7
SSEKSVQPRKLELN	45.88	5.54E6
VASVQQIENR	45.73	8.81E6
NYLEHTKNDYWCGC	45.69	2.19E5
RAIAASSEKSVQPRKLE	45.59	7.3E5
QIENRILA	45.22	1.91E9
KIKKTTPLR	44.75	1.04E7
KKAIIGVSP	44.26	4.32E7
ITNDDKPDVASVQQIE	44.14	4.56E6
ALKRVVLGEG	43.96	8.9E6
EHTKNDYWCGCF	43.87	3.9E5
ALKRVVLGEGVEDY	43.59	4.52E6

EGKGPLKDSLVKKVIILV	43.55	8.56E7
IQELPAVIFEG	42.83	7.39E6
ILAKKTRDYK	42.73	1.01E7
QQIENRILA	42.63	3.18E6
KRVVLGEGVEDYEK	42.13	2.69E5
CGCFITNDDKPDVASVQQIENR	42.08	9.14E5
FIQELPAVIFEG	41.89	5.63E5
KKAIIGVSPEKI	41.86	1.68E7
FITNDDKPDVAS	41.66	7.91E6
IKISFDTWI	41.44	5.49E6
KRVVLGEG	41.09	2.06E7
IQELPAVIF	40.89	5.87E5
KIVLEEFIQEL	40.63	6.33E5
ALKRVVLGE	40.45	2.26E7
ITNDDKPDVASVQQIENRIL	40.18	2.8E6
KKAIIGVSPE	39.93	2.82E6
ELPAVIFE	39.74	5.68E6
KGPLKDSLV	39.51	8.47E5
QIENRILAK	39.50	4.68E7
KRVVLGEGVED	39.49	4.23E6
GVSPEKIVLE	39.32	1.34E6
SVQQIENR	39.18	2.13E7
WCGCFITNDDKPDVASVQQ	39.04	3.75E6
FIQELPAVIFE	37.77	7.04E6
EFIQELPAVIFEG	37.72	1.41E6
TIGWLEESEFSPLRL	37.44	4.47E5
IQELPAVIFEGK	37.03	3.64E6
VASVQQIENRILAK	36.87	7.1E5
NNNPILLS	36.73	4.62E7
VQQIENRILA	35.77	1.22E6
PDVASVQQ	35.65	4.23E6
FITNDDKPDVASVQQIENR	35.59	5.52E6
TKNDYWCGCFI	35.15	2.38E6
GCFITNDDKPDVASVQQ	35.13	1.66E6

NDYWCGCFI	35.10	3.42E5
SSEKSVQPR	35.09	1.51E6
RLGDEAALDYF	34.92	8.29E5
VEDYEKIWN	34.81	2.44E7
ALKRVVLGEGVEDYEKI	34.53	1.19E6
KVSDGSSEIFFK	34.19	1.53E7
EGKGPLKDS	34.16	2.87E7
DSLVKKVIILV	34.05	3.01E6
KRVVLGEGV	33.86	1.54E7
KLELNNNP	33.19	1.27E7
FITNDDKPDV	33.19	1.08E6
CFITNDDKPDVASVQQ	32.75	1.31E6
VNGCRQVWQ	32.69	7.06E5
LGEGVEDYEKIWN	32.48	1.33E6
LLSLKPNPKGEYI	32.45	2.62E6
EGKGPLKDSLVKKVII	32.43	1.07E7
LYDGIRIQADQ	32.27	1.68E6
LNNNPILLS	31.75	8.37E6
ALKRVVLG	31.28	8.42E8
PRKLELNNNPILLS	31.25	1.36E6
KGEYIILS	31.01	3.05E6
KPNPKGEYIILSSLA	30.99	2.02E6
ELNNNPILL	30.59	1.62E6
AVEFDESPEKLWN	30.47	4.15E5
GEGVEDYE	29.83	2.68E7
QELPAVIFE	29.79	1.97E5
GCFITNDDKPDVASVQQIENRILA	29.76	2.67E5
SLVKKVIILV	29.56	4.86E6
KKAIIGVSPEKIVL	28.92	8.48E5
IFEGKGPLK	28.00	4.48E5
EHTKNDYWCGC	27.85	3.7E5
TREIPQEYL	27.79	3.29E5
KISENENILK	27.75	2.67E6
PLKDSLVKK	27.72	2.67E7

RAIAASSEKSVQPRK	27.58	5.87E4
PKGEYIILS	27.28	3.61E7
EDYEKIWNYLEHTKNDYWCG	27.27	8.26E5
SMEHPGAAARLFK	76.25	1.2E6
ECLKDAFVGPTLIAYS	63.23	5.34E6
ATLPTYEEAIARLM	61.03	2.94E6
AAVRDAKEAA	55.61	4.87E6
EGELIPASQIDRLA	53.20	1.75E6
KEASAGKLV	47.62	1.71E6
SAVVADSRGVTVDKMTELR	37.12	4.78E6
SAVVADSRGVT	36.58	2.39E5
RKAGREAGVYM	34.37	7.19E5
ATMKEASAGKLV	31.43	3.37E5
RRAVEGTPFE	29.06	4.44E6
KKASVEIDRKILA	63.58	3.44E6
TALVEKAKAALA	62.52	4.84E7
AAARQNGISYS	47.17	1.92E7
SVEIDRKILA	45.49	3.28E5
QAVIKAGQYA	43.40	1.47E7
GARSRVYR	39.03	7.03E6
SKFINGLK	37.90	2.23E6
VEKAKAALA	36.01	9.5E5
